# A single-cell transcriptomic atlas of all cell types in the brain of 5xFAD Alzheimer mice in response to dietary inulin supplementation

**DOI:** 10.1186/s12915-025-02230-x

**Published:** 2025-05-09

**Authors:** Xiaoyan Wang, Houyu Zhang, Zhou Wan, Xuetong Li, Carlos F. Ibáñez, Meng Xie

**Affiliations:** 1 School of Basic Medical Sciences, Capital Medical University, Beijing, 100069 China; 2 Chinese Institute for Brain Research, Zhongguancun Life Science Park, Beijing, 102206 China; 3 Academy for Advanced Interdisciplinary Studies, Peking University, Beijing, 100871 China; 4 School of Life Sciences, Peking University, Beijing, 100871 China; 5 Peking-Tsinghua Center for Life Sciences, Beijing, 100871 China; 6 PKU-IDG/McGovern Institute for Brain Research, Beijing, 100871 China; 7 Department of Neuroscience, Karolinska Institute, 17165 Stockholm, Sweden; 8 Beijing Key Laboratory of Behavior and Mental Health, School of Psychological and Cognitive Sciences, Peking University, Beijing, 100871 China; 9 Department of Medicine Huddinge, Karolinska Institute, 14183 Stockholm, Sweden

**Keywords:** Alzheimer’s disease, Single-nucleus RNA sequencing, Inulin, Brain

## Abstract

**Background:**

Alzheimer’s disease (AD) is a progressive neurodegenerative disease that is a major threat to the aging population. Due to lack of effective therapy, preventive treatments are important strategies to limit AD onset and progression, of which dietary regimes have been implicated as a key factor. Diet with high fiber content is known to have beneficial effects on cognitive decline in AD. However, a global survey on microbiome and brain cell dynamics in response to high fiber intake at single-cell resolution in AD mouse models is still missing.

**Results:**

Here, we show that dietary inulin supplementation synergized with AD progression to specifically increase the abundance of *Akkermansia muciniphila* in gut microbiome of 5 × Familial AD (FAD) mice. By performing single-nucleus RNA sequencing on different regions of the whole brain with three independent biological replicates, we reveal region-specific changes in the proportion of neuron, astrocyte, and granule cell subpopulations upon inulin supplementation in 5xFAD mice. In addition, we find that astrocytes have more pronounced region-specific diversity than microglia. Intriguingly, such dietary change reduces amyloid-β plaque burden and alleviates microgliosis in the forebrain region, without affecting the spatial learning and memory.

**Conclusions:**

These results provide a comprehensive overview on the transcriptomic changes in individual cells of the entire mouse brain in response to high fiber intake and a resourceful foundation for future mechanistic studies on the influence of diet and gut microbiome on the brain during neurodegeneration.

**Supplementary Information:**

The online version contains supplementary material available at 10.1186/s12915-025-02230-x.

## Background

Alzheimer’s disease (AD) is an irreversible neurodegenerative brain disorder characterized by cognitive decline, memory loss, and language impairment. The histopathological hallmarks of AD include deposition of amyloid-β (Aβ) plaques outside neurons and aggregation of insoluble tau-containing neurofibrillary tangles inside nerve cell bodies, both of which cause neuritic dystrophy and neuronal loss [1]. In response to AD onset, microglia, the innate immune cells of the central nervous system (CNS), undergo microgliosis where they actively proliferate and migrate to protect against AD incidence by restraining the toxic accumulation of Aβ [2]. On the other hand, microgliosis also has been shown to have detrimental effects on neurons, such as synapse engulfment and tau pathology exacerbation [2]. Astrocytes are a morphologically heterogeneous subtype of glia cells that are abundantly present in the CNS. They play many functional roles in the brain, including neuronal trophic support, synaptogenesis, neurovascular coupling, neurotransmitter homeostasis, and maintenance of blood–brain barrier integrity [3]. Reactive astrogliosis in AD is a complex and highly heterogeneous response characterized by astrocyte proliferation and hypertrophy, as well as elevated levels of intermediate filaments, such as glial fibrillar acidic protein (GFAP) and vimentin [4]. Reactive astrocytes have been shown to exhibit different molecular phenotypes in response to lipopolysaccharide-induced systemic neuroinflammation and middle cerebral artery occlusion-induced cerebral ischemia [5], now generally classified as either harmful A1 astrocytes or protective A2 astrocytes [6]. Oligodendrocytes are myelin producing cells in the CNS that ensure the efficient transfer of neuronal signals along the myelinated axons. In the forebrain, oligodendrocytes are derived from several waves of oligodendrocyte progenitor cells (OPCs) that populate the entire embryonic telencephalon [7]. Although AD is considered to mainly influence the gray matter, studies on AD mouse models have demonstrated myelination defects, thereby implicating oligodendrocytes and OPCs [8, 9].

Rapid advances in technologies for single-cell and single-nucleus RNA sequencing (sc- and sn-RNAseq) have allowed comprehensive studies on the diversity and heterogeneity of cell types and subtypes in normal and AD brains. sc-RNAseq of cells from the whole brain of the 5xFAD transgenic mice revealed a unique *Trem2*-enriched microglia population that is involved in restricting neurodegeneration [10]. Moreover, profiling of non-neuronal cells in the hippocampus of wildtype (WT) and 5xFAD mice at single nucleus level identified a subpopulation of disease-associated astrocytes that are in close proximity with Aβ plaques [11]. Comparison of astrocyte subtypes between cortex and hippocampus of WT mice using sc-RNAseq showed clear distinction between the two regions [12]. Finally, integrative analysis of oligodendrocytes in combined datasets of AD and multiple sclerosis mouse models finds three distinct activation states of oligodendrocytes that are immunogenic, survival, and interferon response associated, respectively [13].

Diet is known to be an important risk factor for AD development. Consumption of plant-derived dietary fiber that cannot be digested by mammalian resident gut enzymes has been shown to provide beneficial effects on cognitive performance and memory in both human AD and mouse models [14]. Inulin is a group of indigestible fiber that is found in many vegetables. It can only be metabolized into short-chain fatty acids by the gut commensal bacteria and thereby stimulates their growth and activity [15, 16]. Feeding with inulin-supplemented diet ameliorates neuroinflammation in aged mice [17] and reduces the expression of inflammatory genes in hippocampus of asymptomatic APOE4 transgenic mice [18]. In human, short-term ingestion of oligofructose-enriched inulin can improve mood and memory performance in healthy subjects [19]. How an inulin-rich diet affects the brain is currently unknown. Specifically, the impact of high inulin intake on the transcriptomics of brain cells of AD patients remains to be investigated. In the present study, we set out to obtain a global view on how the transcriptome of individual cells in the entire brain of 5xFAD mice responds to dietary inulin supplementation.

## Results

### Inulin supplementation synergized with AD progression to shape the composition of the gut microbiome

**Fig. 1:**
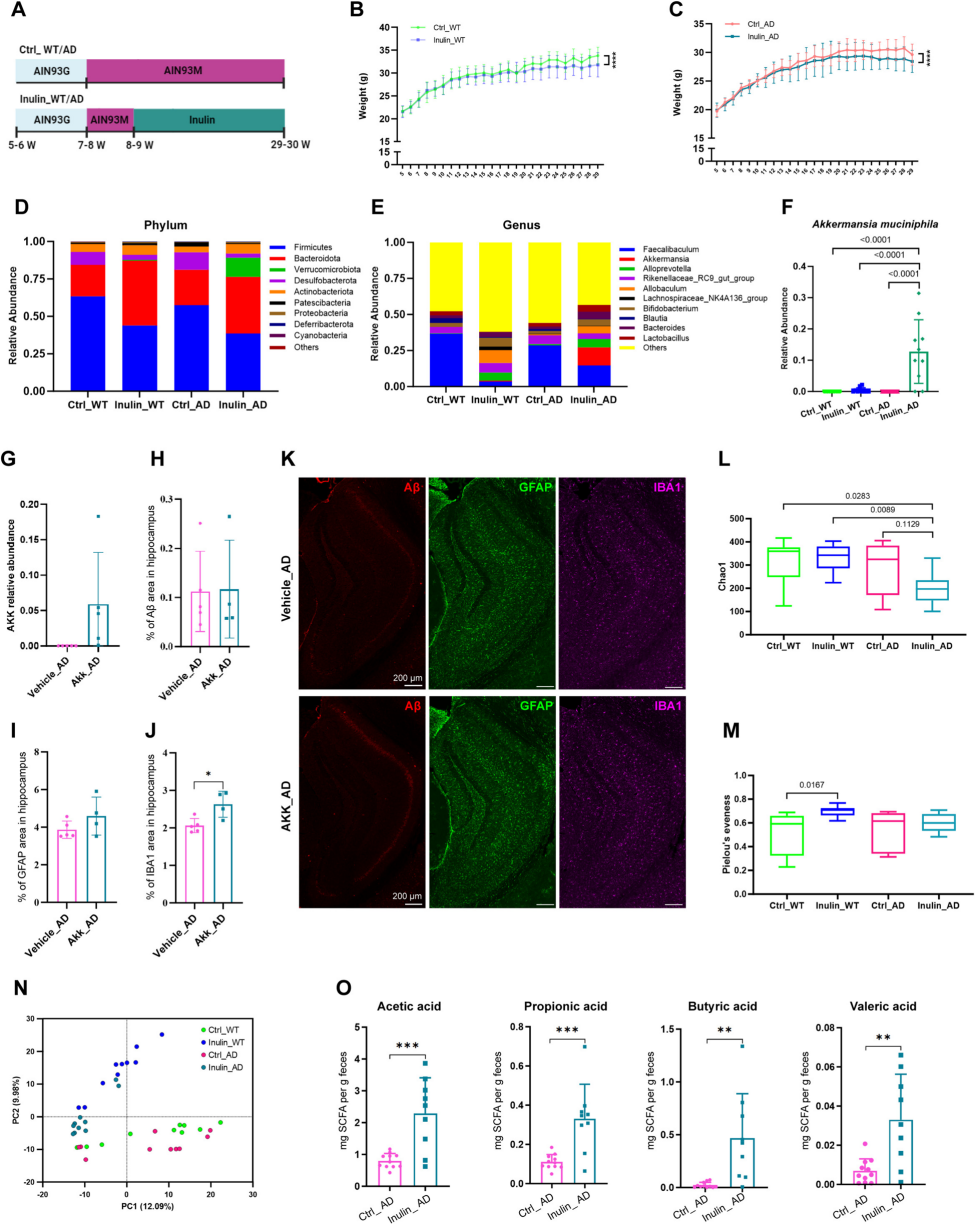
Additive effects of dietary inulin supplementation and AD progression on gut microbiome. **A** Illustration of the feeding scheme of the four diet groups. C57BL/6 J (WT) and 5xFAD (AD) mice were both fed with AIN93G diet from 5–6-week-old to 7–8-week-old. Then, all mice were switched to AIN93M diet. Mice of the Ctrl group were maintained on AIN93M diet, while mice of the inulin group were switched to AIN93M diet supplemented with inulin when they were 8–9-week-old. Mice were collected for analysis at 29–30-week-old. Body weight curves of WT (**B**) and AD (**C**) mice fed with Ctrl or inulin diet. *n* = 10 in all groups. ****, *P* < 0.0001 by two-way ANOVA with Tukey’s multiple comparisons test. Data were presented in mean ± standard deviation (SD). Relative abundance of bacteria at phylum (**D**) and genus (**E**) levels in gut microbiota. *n* = 10 in all groups. **F** Relative abundance of *Akkermansia muciniphila* species. *n* = 10 in all groups. Two-way ANOVA with Sidak’s multiple comparisons test was used to determine statistical significance. Quantification of *Akkermansia muciniphila* abundance (**G**), and the proportion of Aβ (**H**), GFAP (**I**), and IBA1 (**J**) area in hippocampus of 14-week-old 5xFAD mice fed with *Akkermansia muciniphila* (AKK_AD) or medium that does not contain the bacteria (Vehicle_AD) for 4 weeks. Unpaired *t*-test was used to determine statistical significance. *, *P* < 0.05. *n* = 5 in Vehicle_AD group, *n* = 4 in AKK_AD group, represented by a dot in the graphs. Data were presented in mean ± SD. **K** Illustration images of Aβ, GFAP and IBA1 immunostaining in hippocampus. Scale bar, 200 µm. α-Diversity of gut microbiota indicated by Chao1 (**L**) and Pielou’s evenness (**M**) indices. *n* = 10 in all groups. Two-way ANOVA with Tukey’s multiple comparisons test was used to determine statistical significance. **N** PCA analysis of β-diversity of gut microbiota. *n* = 10 in all groups, represented by a dot in the graph. **O** Short-chain fatty acid (SCFA) content in Ctrl_AD and Inulin_AD groups. Unpaired *t*-test was used to determine statistical significance. **, *P* < 0.01; ***, *P* < 0.001. *n* = 10 in Ctrl_AD group, *n* = 9 in Inulin_AD group, represented by a dot in the graphs. Data were presented in mean ± SD. Supporting data values for **B**–**J**, **L**–**O** were included in Additional file 6: Supporting data values. Original images of **K** were included in Additional file 7: original images

We fed 8–9-week-old C57BL/6 J (WT) and 5xFAD mice of the same genetic background [20] with AIN93M diet (Ctrl_WT and Ctrl_AD) or isocaloric AIN93M supplemented with 7.5% (w/w) long-chain inulin (Inulin_WT and Inulin_AD) to 29–30-week-old (Fig. 1A and Additional file 1: Table S1). Animals fed with inulin-supplemented diet showed significantly slower rate of body weight increase than animals fed with the ctrl diet (Fig. 1B–C). At the end of the feeding period, we collected fecal samples from animals of the four groups for 16S sequencing to analyze the composition of their gut microbiome. At the phylum level, inulin supplementation led to increased abundance of Bacteroidota, mainly at the expense of Firmicutes and Desulfobacterota in both WT and AD animals (Fig. 1D). Interestingly, Verrucomicrobiota was almost only present in animals of the Inulin_AD group (Fig. 1D), suggesting that this phylum of Gram-negative bacteria may arise from a synergy of high dietary inulin content and AD progression. At the genus level, inulin supplementation greatly reduced the abundance of *Faecalibaculum* of the Bacillota phylum in both WT and AD animals (Fig. 1E). In contrast, the abundance of *Alloprevotella* (phylum Bacteroidota) and *Allobaculum* (phylum Bacillota) raised dramatically (Fig. 1E). In line with observations at the phylum level, *Akkermansia* genus and *Akkermansia muciniphila* species (phylum Verrucomicrobiota) were specifically enriched in the Inulin_AD group (Fig. 1E–F), supporting the notion that additive effects of inulin and AD progression specifically affect this bacteria species. Next, we fed 14-week-old 5xFAD mice with *Akkermansia muciniphila* (AKK_AD) or culture medium that does not contain the bacteria (Vehicle_AD) twice per week for 4 weeks and collected their feces and hippocampus for analysis at 18-week-old. Similar to the Ctrl_AD animals, *Akkermansia muciniphila* species was only found in the AKK_AD group (Fig. 1G). Using ionized calcium-binding adaptor molecule 1 (IBA1) as a pan-microglial marker, we observed significantly increased microgliosis in AKK_AD animals, but not Aβ plaque burden or astrogliosis (GFAP staining) (Fig. 1H–K), suggesting the limited impact of *Akkermansia muciniphila* supplementation during early phase of AD development.

Assessment of the richness of bacteria species in each group revealed significantly decreased Chao1 index values in the Inulin_AD group compared to the two WT groups (Fig. 1L). A decreasing trend was also observed when comparing to the Ctrl_AD group, although the difference did not reach statistical significance (Fig. 1L). When assessing the evenness in bacteria species abundance using the Pielou’s evenness index, the only difference observed was between Ctrl_WT and Inulin_WT groups, where a significantly higher value indicates more evenly distributed species in the latter (Fig. 1M). These results suggest that inulin supplementation affects different aspects of the α-diversity of gut microbiota of WT and AD animals. Principal component analysis (PCA) of β-diversity showed that animals fed with the same diet were more closely distributed (Fig. 1N), indicating that inulin supplementation has a greater effect on gut microbial communities than AD progression. In line with the role of gut microbiome in metabolizing dietary fiber into short-chain fatty acids, several species were found to be significantly increased in the Inulin_AD group compared to the Ctrl_AD group (Fig. 1O).

### Inulin supplementation reduced microglia proportion and neuronal cholesterol biosynthesis in the forebrain region

**Fig. 2:**
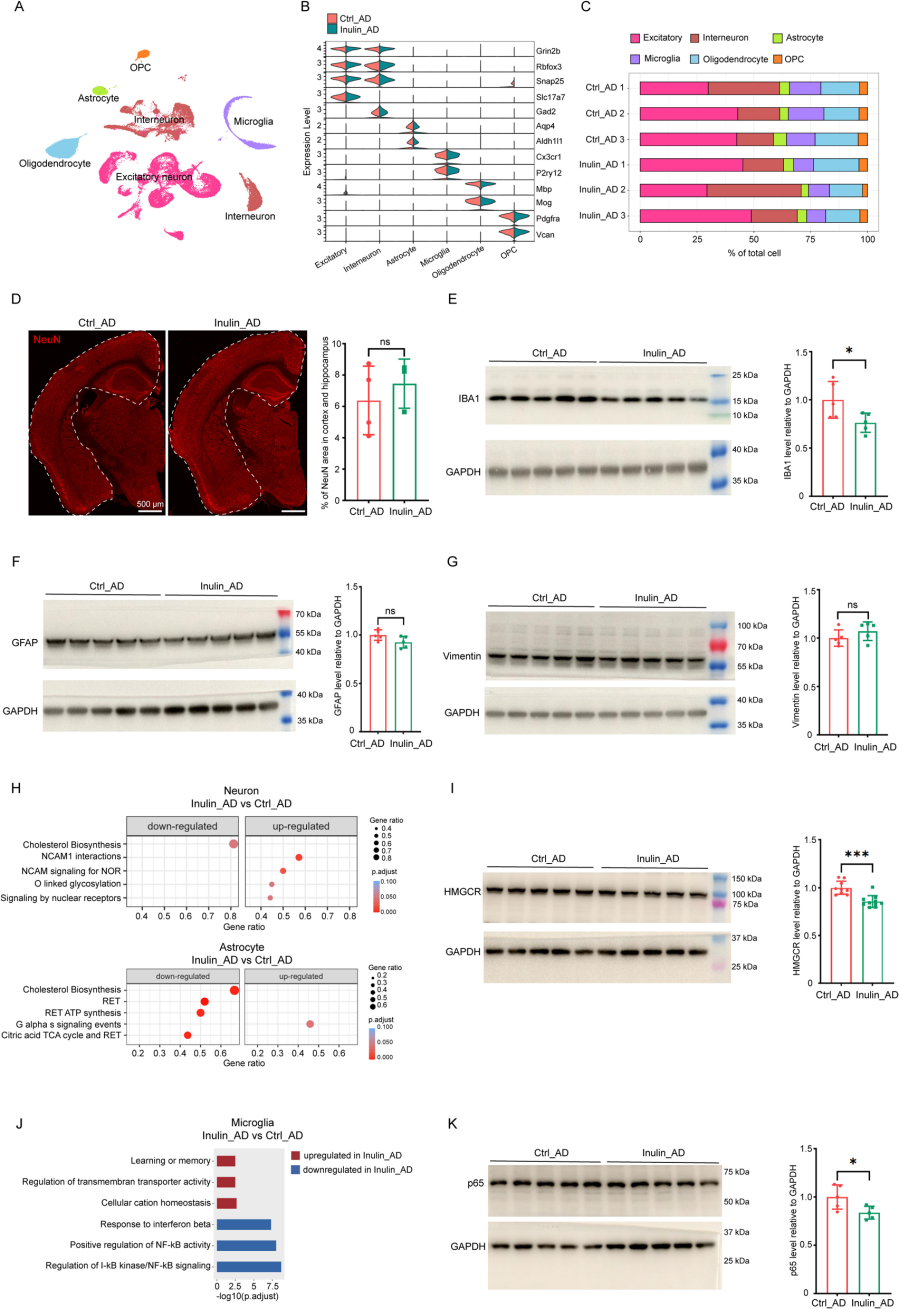
Dietary inulin supplementation altered microglia proportion and neuronal cholesterol biosynthesis in the forebrain region. **A** UMAP of all cell types in the forebrain region of Ctrl_AD and Inulin_AD mice. **B** Violin plot of marker gene expression level of all cell types. **C** Cellular composition comparison between the Ctrl_AD and Inulin_AD groups. **D** Illustrating images and quantification of NeuN immunostaining in cortex and hippocampus (highlighted by white dashed shape in the images) of Ctrl_AD and Inulin_AD animals. Scale bar, 500 µm. Western blots and quantification of IBA1 (**E**), GFAP (**F**), vimentin (**G**), HMGCR (**I**), and RelA (**K**) levels in forebrain homogenates of Ctrl_AD and Inulin_AD animals. In **D**–**G**, **I**, **K**, each dot represents one animal. Unpaired *t*-test was used to determine statistical significance. Data were presented as mean ± SD. For Ctrl_AD and Inulin_AD groups, *n* = 4 and 3 in **D**; *n* = 5 and 5 in **E**–**G**, **K**; *n* = 9 and 10 in **I**. **H** Reactome pathway analysis of DEGs in neuron and astrocyte populations of Ctrl_AD and Inulin_AD animals. **J** GO-BP analysis of DEGs in microglia population of Ctrl_AD and Inulin_AD mice. Supporting data values for **D**–**G**, **I**, **K** were included in Additional file 6: Supporting data values. Original images of **D**–**G**, **I**, **K** were included in Additional file 7: original images

To assess how inulin supplementation affects the transcriptome of cells in different brain regions of the AD brain at single-cell resolution, we separated the entire brain of animals in the Ctrl_AD and Inulin_AD groups into four regions for sn-RNAseq analysis, namely (i) forebrain, containing cerebral cortex, hippocampus, and olfactory bulb; (ii) interbrain, containing thalamus, hypothalamus, striatum and pallidum; (iii) brainstem, containing midbrain, pons, and medulla oblongata; and (iv) cerebellum (Additional file 2: Fig. S1 A). For each feeding condition, we individually sequenced three animals as biological replicates. In the forebrain region, after rigorous filtering (Additional file 2: Fig. S1B) we profiled a total of 7933, 11,234, and 11,788 nuclei for the three animals in the control group and 13,128, 12,826, and 12,756 nuclei for the three animals in the inulin group. In the integrated datasets, clusters corresponding to excitatory neurons (*Slc17a7*^+^), interneurons (*Gad2*^+^), astrocytes (*Aldh1l1*^+^), microglia (*Cx3cr1*^+^), oligodendrocytes (*Mbp*^+^), and oligodendrocyte precursor cells (OPCs) (*Vcan*^+^) were identified (Fig. 2A–B). Proportion of the neuronal populations was comparable between the two diet groups (Fig. 2C), which was further verified by immunostaining in cortex and hippocampus with a NeuN antibody that labels postmitotic neurons (Fig. 2D). Proportion of the microglia cluster was decreased in the Inulin_AD animals (Fig. 2C). Immunoblotting with an IBA1 antibody against microglia-specific calcium-binding protein revealed similar decreasing trend in forebrain homogenates (Fig. 2E). Protein levels of astrocyte-specific markers, GFAP, and vimentin were similar between the two diet groups (Fig. 2F–G), which is in line with their comparable cellular proportions (Fig. 2C). Gene Set Enrichment Analysis (GSEA) of the differentially expressed genes (DEGs) in the neuron and astrocyte populations showed that cholesterol biosynthesis was the top downregulated pathway in the Inulin_AD animals (Fig. 2H). Consistently, the protein level of HMGCR, a reductase that catalyzes the rate-limiting reaction in cholesterol synthesis, was significantly reduced in the forebrain homogenates of Inulin_AD animals (Fig. 2I). Gene ontology (GO) analysis of the DEGs in the microglia population revealed downregulation of several NF-κB signaling pathways in the Inulin_AD animals (Fig. 2J), partly due to the reduced p65 protein level (Fig. 2K). No difference was observed in sub-cluster proportions of neuron, microglia, astrocyte, oligodendrocyte, or OPC populations between the two groups (Additional file 3: Fig. S2).

### Inulin supplementation altered the proportion of astrocyte subpopulations in the interbrain region

**Fig. 3:**
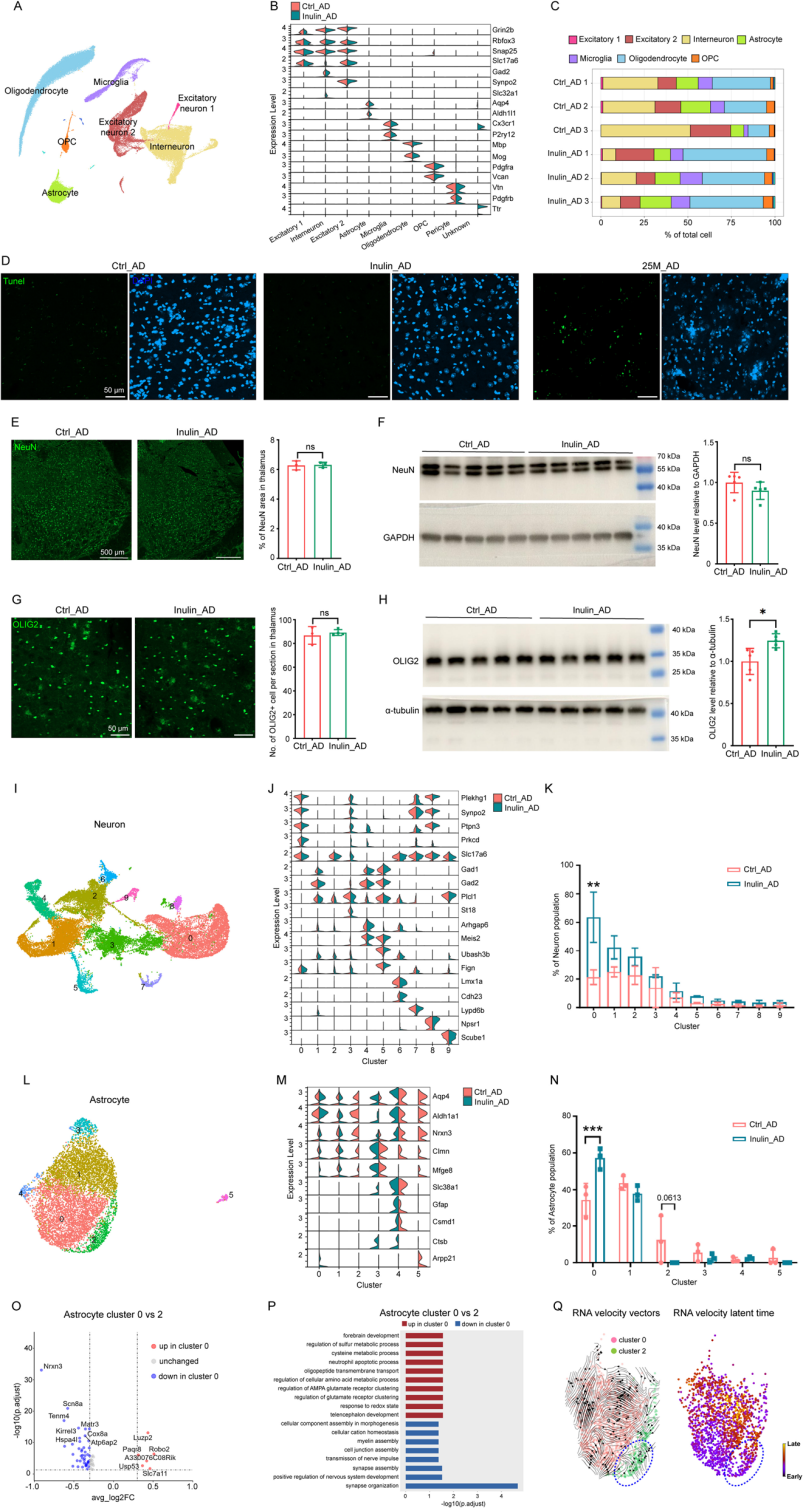
Dietary inulin supplementation altered the proportion of astrocyte subpopulations in the interbrain region. **A** UMAP of all cell types in the interbrain region of Ctrl_AD and Inulin_AD mice. **B** Violin plot of marker gene expression level of all cell types. **C** Cellular composition comparison between the Ctrl_AD and Inulin_AD groups. Illustrating images and quantification of Tunel (**D**), NeuN (**E**), and OLIG2 (**G**) staining in the thalamus of Ctrl_AD and Inulin_AD mice. Scale bar, 50 µm in **D**, **G**; scale bar, 500 µm in **E**. Western blots and quantification of NeuN (**F**) and OLIG2 (**H**) levels in the thalamus homogenates of Ctrl_AD and Inulin_AD mice. UMAPs of neuron (**I**) and astrocyte (**L**) subtypes in the interbrain region of Ctrl_AD and Inulin_AD mice. Violin plots of marker gene expression level in neuron (**J**) and astrocyte (**M**) subtypes. Comparison of neuron (**K**) and astrocyte (**N**) composition. In **E**–**H**, each dot represents one animal. Unpaired *t*-test was used to determine statistical significance. In **K**, **N**, two-way ANOVA with Sidak’s multiple comparisons test was used to determine statistical significance. Data were presented as mean ± SD. For Ctrl_AD and Inulin_AD groups, *n* = 3 and 3 in **E**, *n* = 5 and 5 in **F**, **H**, *n* = 3 and 4 in **G**. **O** Volcano plot of DEGs of cluster 0 astrocytes against cluster 2. Red dots represent the genes that were upregulated in cluster 0 compared to cluster 2. Blue dots represent the genes that were downregulated in cluster 0. Absolute log2 fold change greater than 0.3 and *P* < 0.05 were used as selection criteria. **P** GO analysis of DEGs in astrocyte cluster 0 and 2. Pathways that were upregulated in cluster 0 compared to cluster 2 were shown in red. Pathways that were downregulated in cluster 0 were shown in blue. **Q** Analysis of transcriptional trajectories in cluster 0 and 2 astrocytes. Arrows represent the predicted transcriptional flow based on RNA velocity analysis of spliced versus unspliced mRNA transcripts. Cluster 2 cells that flow towards cluster 0 were highlighted by the blue dashed circle. Heat map illustrating the latent splice time of cluster 0 and 2 astrocytes, based on splicing kinetics derived from the RNA velocity model. Color of each dot represents the level of progression of each cell along the differentiation trajectories defined by the RNA velocity model. Supporting data values for **E**–**H** were included in Additional file 6: Supporting data values. Original images of **D**–**H** were included in Additional file 7: original images

In the interbrain region that contained mostly thalamus and hypothalamus, a total of 12,897, 8470, and 13,240 nuclei for the three animals in the control group and 10,232, 8884, and 8846 nuclei for the three animals in the inulin group were analyzed after filtering (Additional file 2: Fig. S1 C). Clusters of excitatory neurons (*Slc17a7*^+^), interneurons (*Gad2*^+^), astrocytes (*Aldh1l1*^+^), microglia (*Cx3cr1*^+^), oligodendrocytes (*Mbp*^+^), OPCs (*Vcan*^+^), and pericytes (*Vtn*^+^) were identified (Fig. 3A–B). Unexpectedly, a dramatic decrease in the proportion of interneuron population was observed in the Inulin_AD animals, accompanied by a reciprocal increase in the proportion of oligodendrocytes (Fig. 3C). Similar level of Tunel staining in thalamus was observed between the two groups (Fig. 3D), indicating that the proportional differences were not due to cell apoptosis. Surprisingly, immunostaining in thalamus (Fig. 3E) and immunoblotting in interbrain homogenates (Fig. 3F) showed comparable protein levels of NeuN between the two groups. Such inconsistency between analyses performed at the transcriptomic and protein levels may be caused by spatial and temporal fluctuations of mRNA transcripts and efficiency of protein biosynthesis as previously discussed [21]. Unlike the neurons, Inulin_AD animals showed consistent increase of OLIG2 (an oligodendrocyte-specific antibody) protein level in both thalamus and the interbrain homogenates (Fig. 3G–H).

After zooming into the neuronal subpopulations, we found a significant increase in the proportion of a subset of excitatory neuron in the Inulin_AD animals that specifically expressed *Prkcd*, a marker gene of neurons in the central nucleus of the amygdala (CeA) (cluster 0) (F ig. 3I–K). Re-clustering of the astrocyte population revealed a significant increase in the proportion of cluster 0 of the Inulin_AD group, whereas cluster 2 was completely lost (Fig. 3L–N). GO analysis of the DEGs of the two clusters showed upregulation of several metabolic pathways and downregulation of pathways related to neuronal structure assembly in cluster 0 astrocytes (Fig. 3O–P). To assess the major transcriptional trajectories between these two astrocyte subpopulations, we applied RNA velocity to predict their temporal gene expression dynamics based on the spliced and unspliced RNA transcripts [22]. We noticed that some RNA velocity vectors of cluster 2 cells pointed in the direction of cluster 0, while most of the cluster 0 vectors pointed away from cluster 2 (Fig. 3Q). Latent time, an approximation of the real time that cells experience as they differentiate, indicated that most of the cluster 0 cells that flowed towards cluster 2 were at relatively early differentiation stage (Fig. 3Q). Given the reciprocal changes in the proportion of cluster 0 and 2 under the two diet conditions, a potential conversion of cluster 2 to cluster 0 astrocytes may exist upon inulin supplementation. Finally, further clustering of microglia, oligodendrocyte, and OPC populations did not show any significant differences in any of the sub-cluster proportions between the two diets (Additional file 4: Fig. S3).

### Inulin supplementation altered the proportion of astrocyte subpopulations in brainstem

**Fig. 4:**
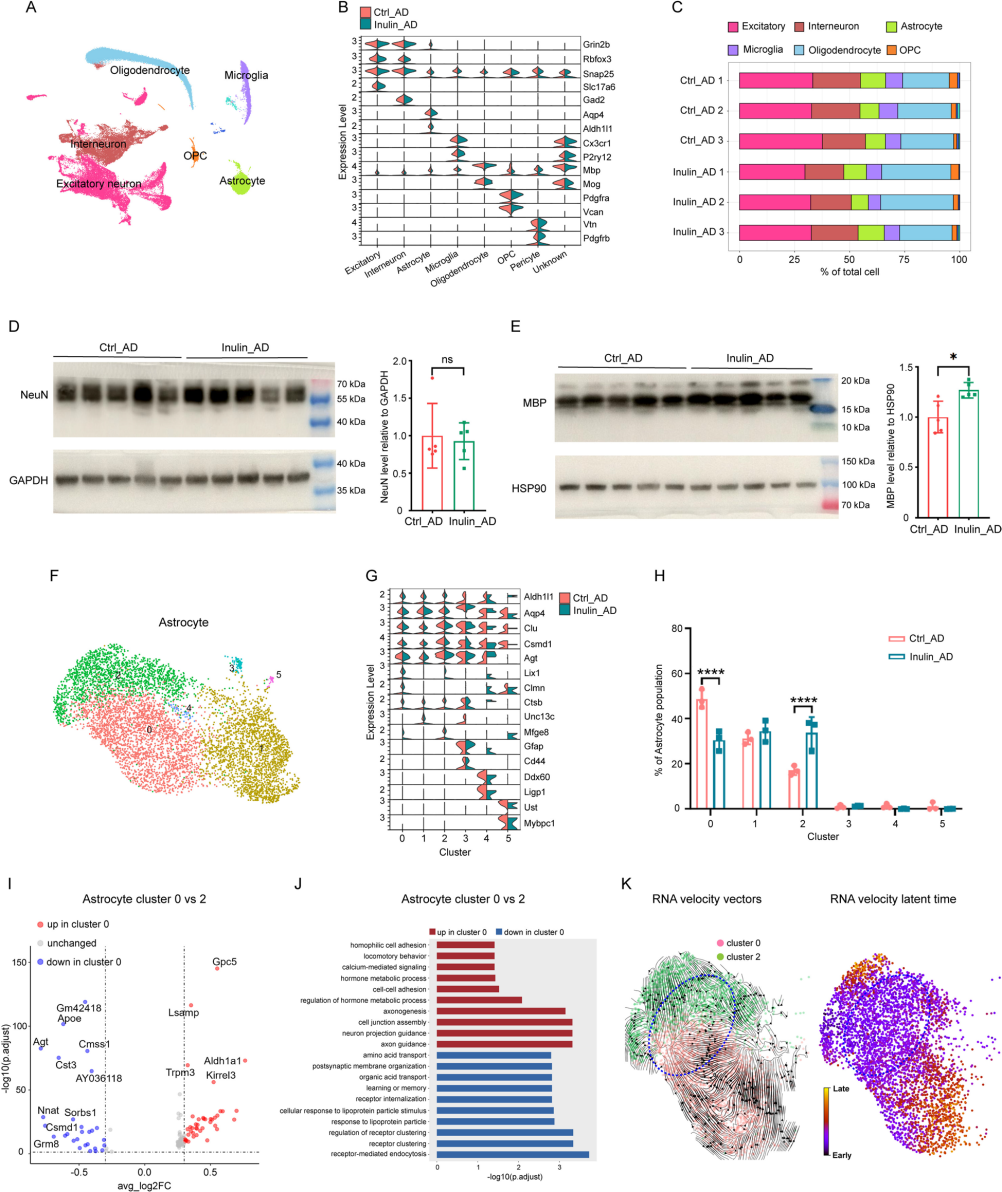
Dietary inulin supplementation altered the proportion of astrocyte subpopulations in brainstem. **A** UMAP of all cell types in the brainstem of Ctrl_AD and Inulin_AD mice. **B** Violin plot of marker gene expression level of all cell types. **C** Cellular composition comparison between the Ctrl_AD and Inulin_AD groups. Western blots and quantification of NeuN (**D**) and MBP (**E**) levels in the brainstem homogenates of Ctrl_AD and Inulin_AD mice. Each dot represents one animal. Unpaired *t*-test was used to determine statistical significance. Data were presented as mean ± SD. *n* = 5 for both groups. **F** UMAP of astrocyte subtypes in the brainstem of Ctrl_AD and Inulin_AD mice. **G** Violin plot of marker gene expression level in astrocyte subtypes. **H** Comparison of astrocyte composition. Each dot represents one animal. Two-way ANOVA with Sidak’s multiple comparisons test was used to determine statistical significance. Data were presented as mean ± SD. **I** Volcano plot of DEGs of cluster 0 astrocytes against cluster 2. Red dots represent the genes that were upregulated in cluster 0 compared to cluster 2. Blue dots represent the genes that were downregulated in cluster 0. Absolute log2 fold change greater than 0.3 and *P* < 0.05 were used as selection criteria. **J** GO analysis of DEGs in astrocyte cluster 0 and 2. Pathways that were upregulated in cluster 0 compared to cluster 2 were shown in red. Pathways that were downregulated in cluster 0 were shown in blue. **K** Analysis of transcriptional trajectories in cluster 0 and 2 astrocytes. Arrows represent the predicted transcriptional flow based on RNA velocity analysis of spliced versus un-spliced mRNA transcripts. Cells at the border between cluster 0 and 2 were highlighted by the blue dashed circle. Heat map illustrating the latent splice time of cluster 0 and 2 astrocytes, based on splicing kinetics derived from the RNA velocity model. Color of each dot represents the level of progression of each cell along the differentiation trajectories defined by the RNA velocity model. Supporting data values for **D**–**E** were included in Additional file 6: Supporting data values. Original images of **D**–**E** were included in Additional file 7: original images

In brainstem, a total of 11,900, 13,543, and 11,084 nuclei for the three animals in the control group and 14,534, 11,965, and 11,293 nuclei for the three animals in the inulin group were analyzed after filtering (Additional file 2: Fig. S1D). Clusters of excitatory neurons (*Slc17a6*^+^), interneurons (*Gad2*^+^), astrocytes (*Aqp4*^+^), microglia (*Cx3cr1*^+^), oligodendrocytes (*Mbp*^+^), OPCs (*Vcan*^+^), and pericytes (*Vtn*^+^) were identified (Fig. 4A–B). Proportion of the neuronal populations was similar between the two diet groups (Fig. 4C), which was further confirmed by immunoblotting of the brainstem homogenates with a NeuN antibody (Fig. 4D). Proportion of the oligodendrocyte cluster appeared to be increased in the Inulin_AD animals (Fig. 4C), which is in line with the immunoblotting against an oligodendrocyte-specific protein MBP (Fig. 4E). Re-clustering of the astrocyte population revealed a significant decrease in the proportion of *Lix1*&*Clmn*-expressing sub-cluster (cluster 0) and a significant increase in the *Mfge8*-expressing sub-cluster (cluster 2) in the Inulin_AD group (Fig. 4F–H). GO analysis of the DEGs of the two clusters revealed upregulation of neurogenesis pathways and downregulation of pathways related to nutrient transport and receptor mobilization in cluster 0 astrocytes in the Inulin_AD group (F ig. 4I–J). RNA velocity analysis revealed opposite flow directions of cluster 0 and 2 cells at their border and comparable latent splice time (Fig. 4K), suggesting against an interconversion between these two astrocyte subpopulations. We did not find marker genes with high expression level that were specifically expressed in cluster 0 or 2 astrocytes (Fig. 4G). In line with this notion, immunostaining with antibodies against two of the highly expressed marker genes in the two clusters (AQP4 and CLU) revealed signal overlaps in both diet groups (Additional file 5: Fig. S4A). No significant differences were observed between the two groups after re-clustering the neuron, microglia, oligodendrocyte, and OPC populations (Additional file 5: Fig. S4B–M).

### Inulin supplementation altered the proportion of granule cell subpopulations in cerebellum

**Fig. 5:**
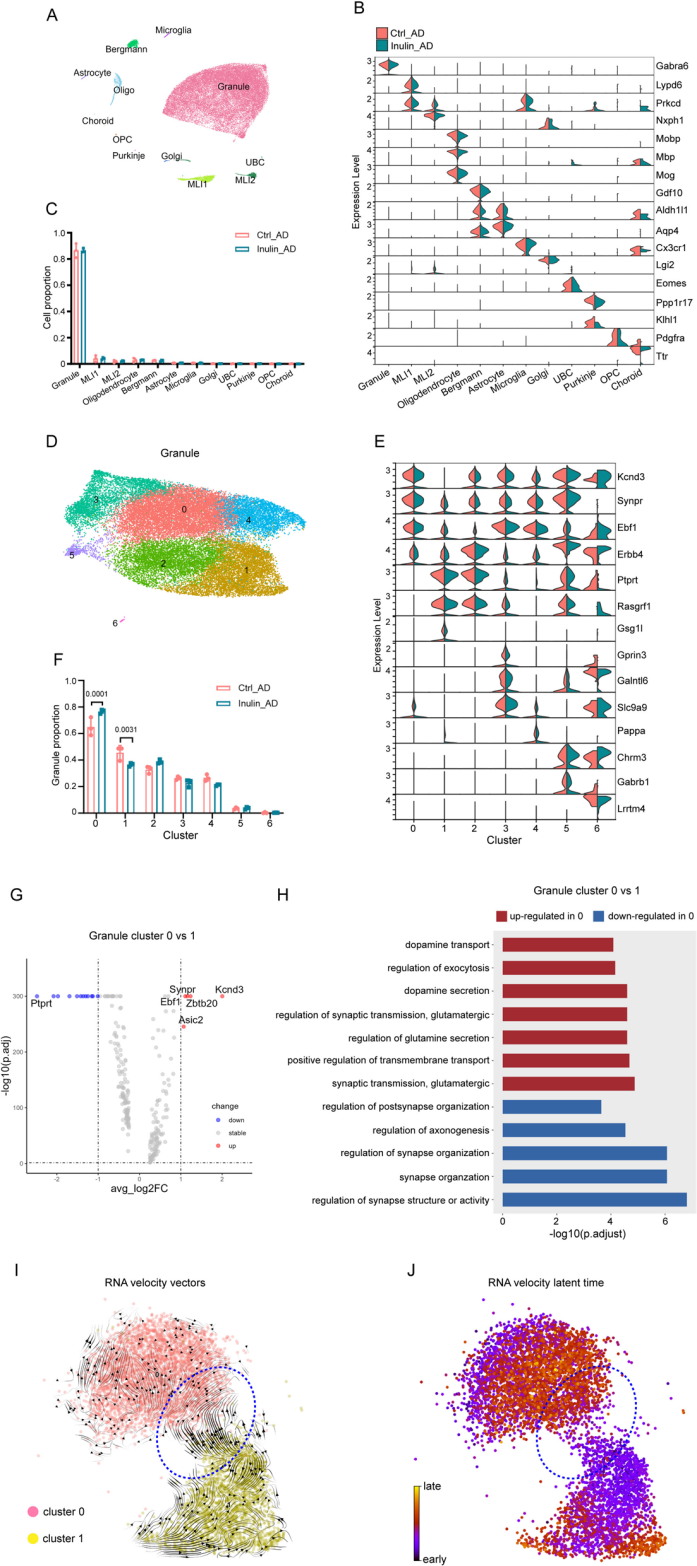
Dietary inulin supplementation altered the proportion of granule cell subpopulations in cerebellum. UMAP of all cell types (**A**) and granule cells (**D**) in the cerebellum of Ctrl_AD and Inulin_AD mice. Violin plot of marker gene expression level of all cell types (**B**) and granule cells (**E**). Cell proportion comparison of each cell type (**C**) and granule cell subtypes (**F**) between the Ctrl_AD and Inulin_AD groups. Each dot represented one independently sequenced mouse. Two-way ANOVA with Sidak’s multiple comparisons test was used to determine statistical significance. Data were presented in mean ± SD. **G** Volcano plot of DEGs of cluster 0 granule cells against cluster 1. Red dots represent the genes that were upregulated in cluster 0 compared to cluster 1. Blue dots represent the genes that were downregulated in cluster 0. Absolute log2 fold change greater than 0.3 and *P* < 0.05 were used as selection criteria. **H** GO analysis of DEGs in granule cell cluster 0 and 1. Pathways that were upregulated in cluster 0 compared to cluster 1 were shown in red. Pathways that were downregulated in cluster 0 were shown in blue. **I** Analysis of transcriptional trajectories in cluster 0 and 1 granule cells. Arrows represent the predicted transcriptional flow based on RNA velocity analysis of spliced versus un-spliced mRNA transcripts. Cells at the border between cluster 0 and 1 were highlighted by the blue dashed circle. **J** Heat map illustrating the latent splice time of cluster 0 and 1 granule cells, based on splicing kinetics derived from the RNA velocity model. Color of each dot represents the level of progression of each cell along the differentiation trajectories defined by the RNA velocity model

In cerebellum, we analyzed 5111, 5796, and 5280 nuclei for the three animals in the Ctrl_AD group and 6414, 5138, and 6030 nuclei for the three animals in the Inulin_AD group after filtering (Additional file 2: Fig. S1E). Clusters of granule cells (*Gabra6*^+^), MLI1 interneurons (*Lypd6*^+^), MLI2 interneurons (*Nxph1*^+^), oligodendrocytes (*Mbp*^+^), Bergmann cells (*Gdf10*^+^), astrocytes (*Aqp4*^+^), microglia (*Cx3cr1*^+^), Golgi cells (*Lgi2*^+^), unipolar brush cells (*Eomes*^+^), Purkinje cells (*Ppp1r17*^+^), OPCs (*Pdgfra*^+^), and choroid plexus cells (*Ttr*^+^) were identified (Fig. 5A–B). As expected, most of the identified cells were granule cells (Fig. 5C). Their re-clustering revealed reciprocal changes in the proportion of *Ebf1*^+^&*Rasgrf1*^−^ (cluster 0) and *Rasgrf1*^+^&*Kcnd3*^−^ (cluster 1) subpopulations (Fig. 5D–F). DEG-based GO comparison of these two subpopulations showed that cluster 0 showed enrichment in pathways involved in dopamine secretion and transport, as well as glutamatergic synaptic transmission, while had downregulated pathways related to synapse organization (Fig. 5G–H). RNA velocity analysis showed unidirectional flow of cluster 1 cells towards cluster 0 at the border (Fig. 5I) and clear separation in latent time (Fig. 5J), suggesting possible conversion of cluster 1 granule cells into cluster 0 upon inulin supplementation.

### Astrocytes show greater region-specific diversity than microglia

**Fig. 6:**
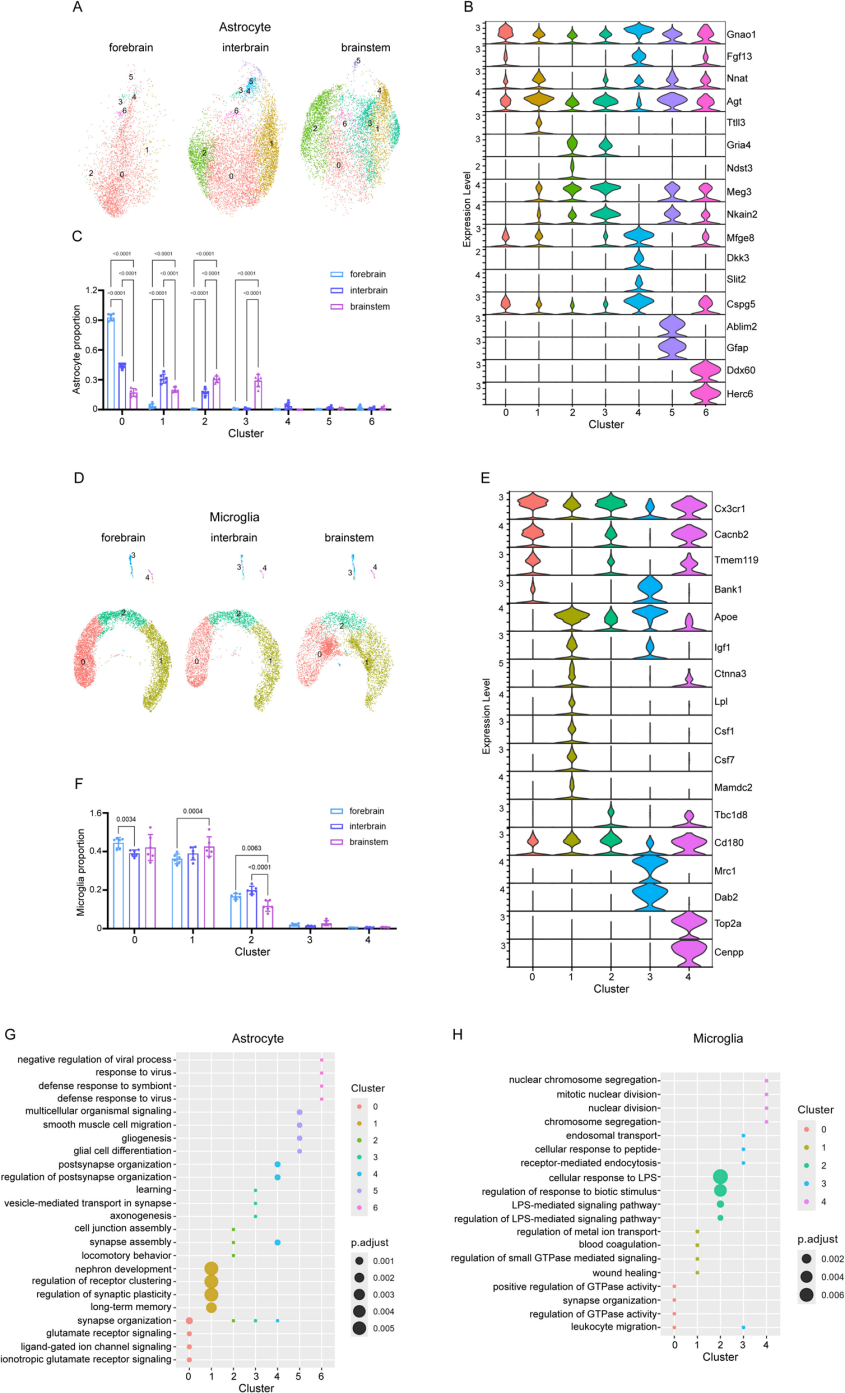
Astrocyte subpopulations distributed in a region-specific manner. UMAP of astrocytes (**A**) and microglia (**D**) in the forebrain, interbrain, and brainstem of Ctrl_AD and Inulin_AD mice. Violin plot of marker gene expression level of astrocytes (**B**) and microglia (**E**). Cell proportion comparison of astrocyte (**C**) and microglia (**F**) subtypes among the three regions. Each dot represented one independently sequenced mouse. Two-way ANOVA with Tukey’s multiple comparisons test was used to determine statistical significance. Data were presented in mean ± SD. GO analysis of DEGs in astrocyte (**G**) and microglia (**H**) subpopulations. LPS, lipopolysaccharide

Next, we merged the astrocytes from forebrain, interbrain, and brainstem regions of Ctrl_AD and Inulin_AD mice to investigate their spatial distribution pattern. Cerebellum was not included due to very limited number of astrocytes identified in the datasets. Forebrain region was mostly occupied by the *Gnao1*&*Fgf13*-expressing subpopulation (cluster 0) that was enriched with pathways related to neuronal development, while its occupancy in the other two brain regions was significantly lower (Fig. 6A–C, G). In addition to cluster 0, the *Nnat*-expressing subpopulation (cluster 1) that had upregulated lipid metabolism processes and the *Gria4*&*Ndst3*-expressing subpopulation (cluster 2) that was involved in neurotransmitter and ion transport were also found to be the major constituents of astrocytes in the interbrain and brainstem regions (Fig. 6A–C, G). The *Mfge8*-expressing subpopulation (cluster 3) that was highlighted with synapse function-related pathways was only found in brainstem, occupying about 30% of the total astrocyte population (Fig. 6A–C, G). These results indicate that astrocyte subtypes with different functions have specific distribution patterns in different brain regions. In contrast, the microglia subpopulations exhibited much less variations among the three brain regions (Fig. 6D–F, H). The *Tmem119*^+^ homeostatic sub-cluster (cluster 0) was slightly lower in the interbrain region compared to forebrain, while the *Apoe*^+^ disease-associated sub-cluster (cluster 1) was higher in brainstem than forebrain (Fig. 6D–F, H). Another major microglia subpopulation that expressed both *Tmem119* and *Apoe* (cluster 2) was found to be higher in the forebrain and interbrain regions (Fig. 6D–F, H). Notably, this microglia subpopulation that is likely to be in an intermediate state was not identified when comparing the microglia of the same region between the two diets (Additional file 3: Fig. S2D–E and Additional file 4: Fig. S3A–B), suggesting that regional difference has more pronounced impact on microglia diversity than dietary difference.

### Inulin supplementation reduced overall Aβ plaque burden and microgliosis in the forebrain region

**Fig. 7:**
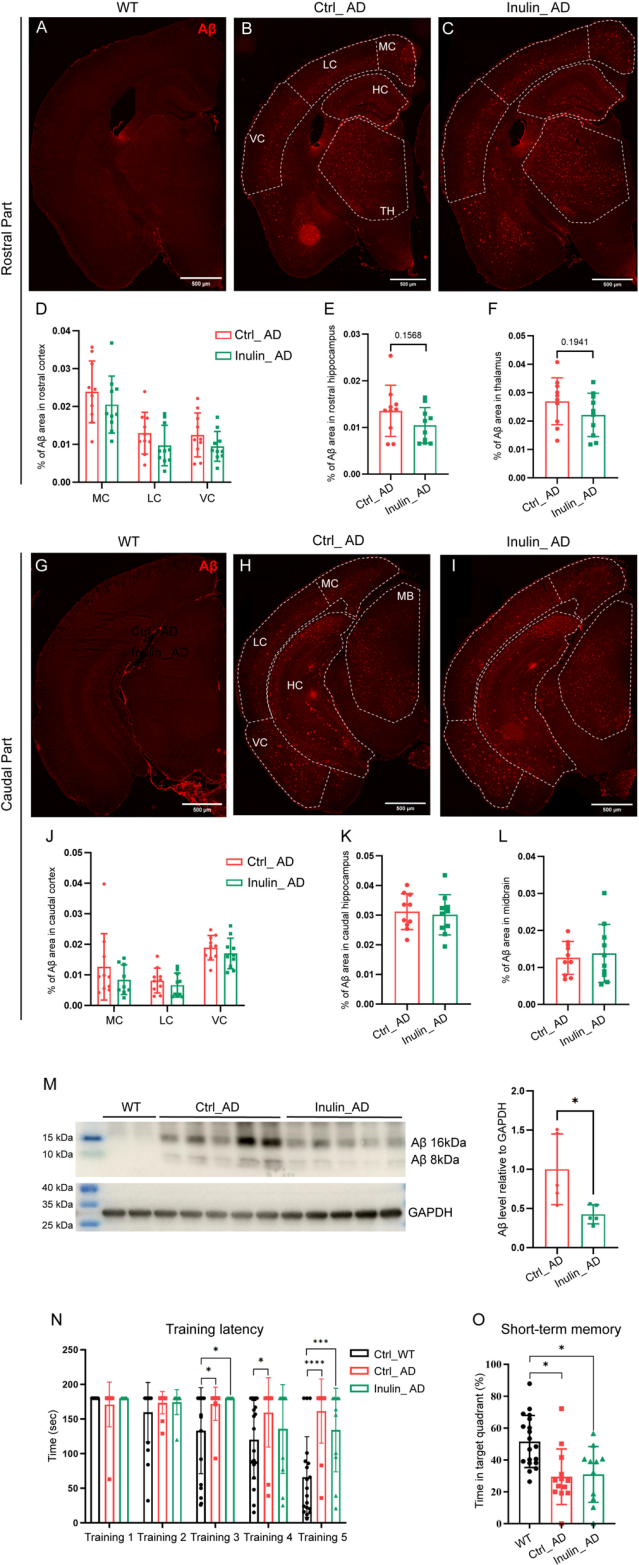
Dietary inulin supplementation reduced forebrain Aβ plaque burden. Illustration of Aβ plaque deposition by 6E10 antibody against human Aβ peptide in rostral (**A**–**C**) and caudal (**G**–**I**) parts of the brain of WT (**A**, **G**), Ctrl_AD (**B**, **H**), and Inulin_AD (**C**, **I**) mice. MC, medial cortex; LC, lateral cortex; VC, ventral cortex; HC, hippocampus; TH, thalamus; MB, midbrain. Scale bar, 500 µm. **D**–**F**, **J**–**L** Quantification of the percentage of Aβ area in different brain regions. Comparisons in different regions of cortex were performed by two-way ANOVA with Sidak’s multiple comparisons test (**D**, **J**). Unpaired *t*-test was used to assess the other brain regions (**E**–**F**, **K**–**L**). *P* values that were less than 0.2 were indicated. *n* = 10 in all groups, represented by a dot in the graphs. Data were presented in mean ± SD. **M** Western blot and quantification of Aβ level in the forebrain region of Ctrl_AD and Inulin_AD mice. Integral density of Aβ 16 and 8 kDa bands was added for quantification. Unpaired *t*-test was used to determine statistical significance. *, *P* < 0.05. *n* = 5 in both groups, represented by a dot in the graphs. Data were presented in mean ± SD. **N**–**O** Training latency (time to find the escape tunnel) and percentage of time spent in the target quadrant of the Barnes maze test 3 h after the last training. Data were presented in mean ± SEM. *n* = 19, 13, and 11 mice for the Ctrl_WT, Ctrl_AD, and Inulin_AD groups, respectively, represented by a dot in the histograms. Two-way ANOVA with Sidak’s multiple comparisons test and one-way ANOVA with Sidak’s multiple comparisons test were used in **M** and **N**, respectively. Supporting data values for **D**–**F**, **J**–**K**, **L**–**O** were included in Additional file 6: Supporting data values. Original images of **A**–**C**, **G**–**I**, **M** were included in Additional file 7: original images

Next, we assessed Aβ plaque burden in different brain regions of Ctrl_WT, Ctrl_AD, and Inulin_AD animals by immunohistochemistry. No significant difference was observed for Aβ plaque burden in any of the examined brain regions, besides a decreasing trend in the rostral hippocampus and thalamus (Fig. 7A–L). However, western blot analysis on forebrain homogenate revealed significant decrease in Aβ protein level in Inulin_AD animals (Fig. 7M), suggesting a general reduction of Aβ plaque burden in this region. We also performed the Barnes maze test to assess the impact of inulin supplementation on spatial learning and memory. During the training phase, Ctrl_WT animals showed progressively decreasing latencies in finding the escape tunnel, whereas the Ctrl_AD animals did not show obvious reduction in training latency (Fig. 7N). Compared to the Ctrl_AD animal, the average training latency of Inulin_AD animal was reduced by about 10% during the fourth and fifth training, which was still significantly longer than the Ctrl_WT animal (Fig. 7N). Probe test was conducted 3 h after the last training to assess short-term memory of the escape tunnel location. While Ctrl_WT animals spent over half of the probe time in the target quadrant, Ctrl_AD and Inulin_AD animals performed no difference from random probability (around 25% probe time in the target quadrant) (Fig. 7O).

**Fig. 8:**
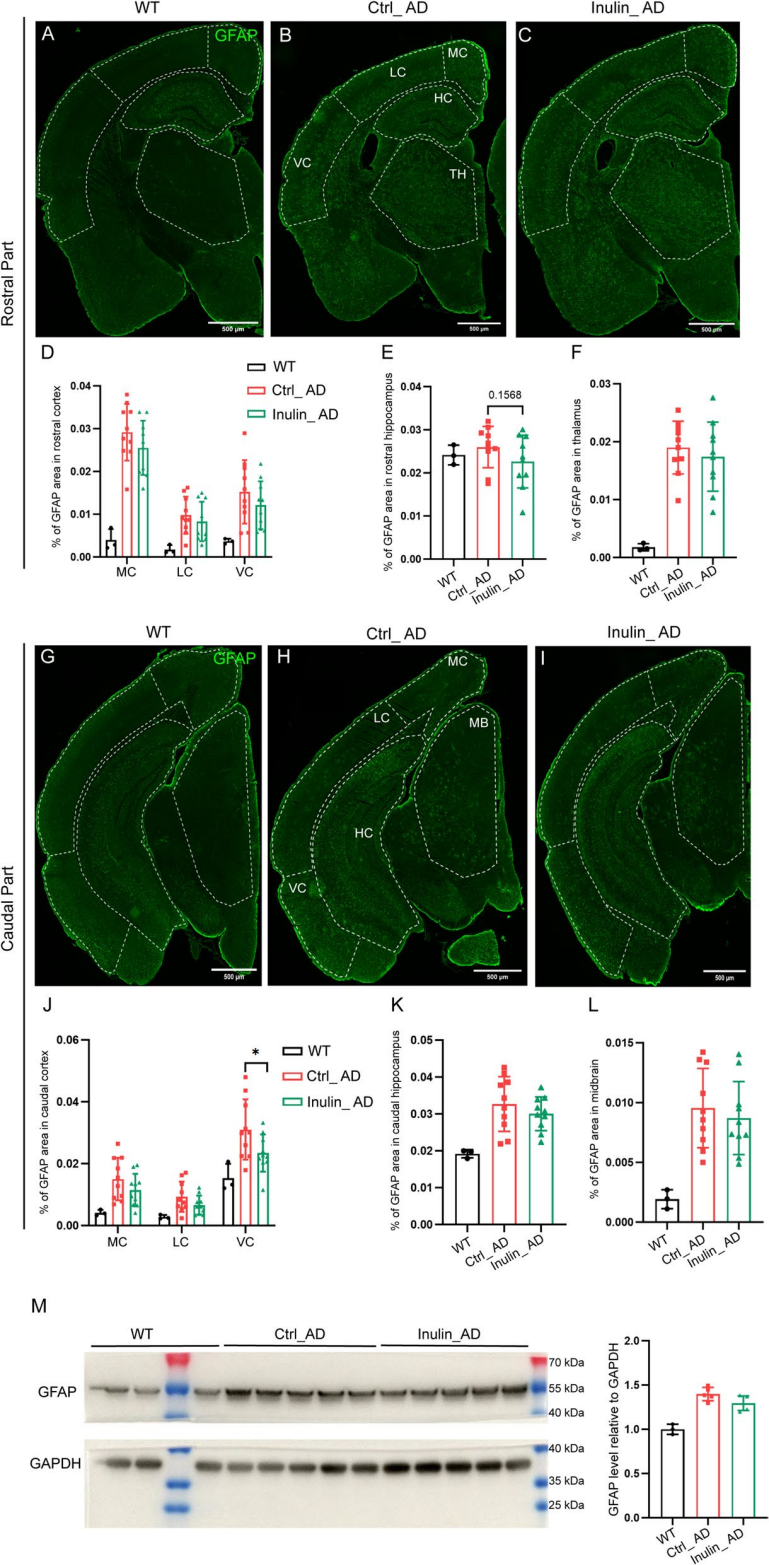
Dietary inulin supplementation reduced astrogliosis in caudal part of the ventral cortex. Illustration of reactive astrocyte distribution by GFAP antibody in rostral (**A**–**C**) and caudal (**G**–**I**) parts of the brain of WT (**A**, **G**), Ctrl_AD (**B**, **H**), and Inulin_AD (**C**, **I**) mice. MC, medial cortex; LC, lateral cortex; VC, ventral cortex; HC, hippocampus; TH, thalamus; MB, midbrain. Scale bar, 500 µm. **D**–**F**, **J**–**L** Quantification of the percentage of GFAP area in different brain regions. Comparisons in different regions of cortex were performed by two-way ANOVA with Sidak’s multiple comparisons test (**D**, **J**). Unpaired *t*-test was used to assess the other brain regions (**E**–**F**, **K**–**L**). *P* values that were less than 0.2 were indicated. *n* = 3 in WT group, *n* = 10 in Ctrl_AD and Inulin_AD groups, represented by a dot in the graphs. Data were presented in mean ± SD. **M** Western blot and quantification of GFAP level in the forebrain region of WT, Ctrl_AD, and Inulin_AD mice. Unpaired *t*-test was used to determine statistical significance between Ctrl_AD and Inulin_AD groups. *n* = 3 in WT group, *n* = 5 in Ctrl_AD and Inulin_AD groups, represented by a dot in the graphs. Data were presented in mean ± SD. Supporting data values for **D**–**F**, **J**–**K**, **L**–**M** were included in Additional file 6: Supporting data values. Original images of **A**–**C**, **G**–**I**, **M** were included in Additional file 7: original images

**Fig. 9:**
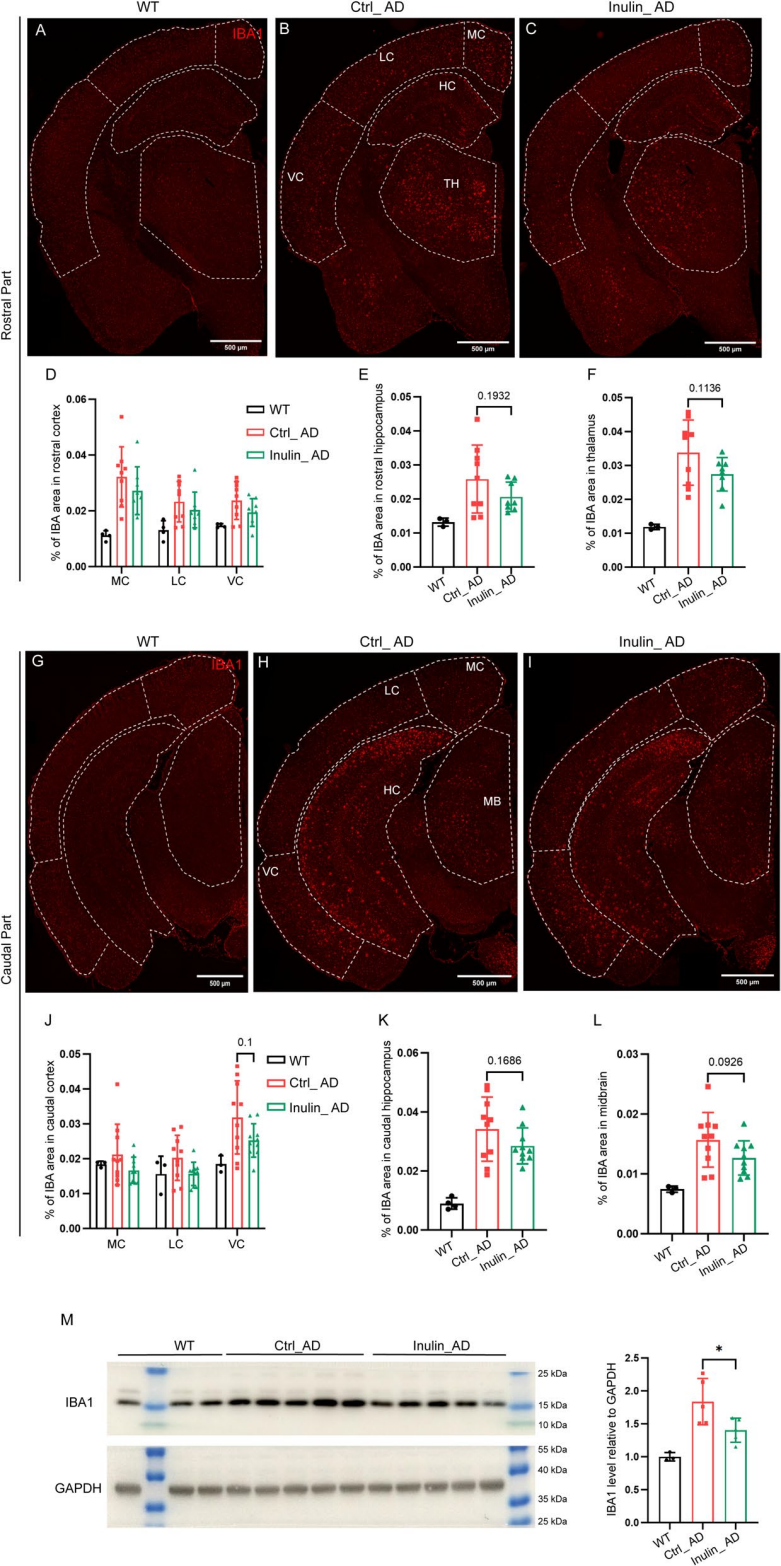
Dietary inulin supplementation reduced forebrain microgliosis. Illustration of microglia distribution by IBA1 antibody in rostral (**A**–**C**) and caudal (**G**–**I**) parts of the brain of WT (**A**, **G**), Ctrl_AD (**B**, **H**), and Inulin_AD (**C**, **I**) mice. MC, medial cortex; LC, lateral cortex; VC, ventral cortex; HC, hippocampus; TH, thalamus; MB, midbrain. Scale bar, 500 µm. **D**–**F**, **J**–**L** Quantification of the percentage of IBA1 area in different brain regions. Comparisons in different regions of cortex were performed by two-way ANOVA with Sidak’s multiple comparisons test (**D**, **J**). Unpaired *t*-test was used to assess the other brain regions (**E**–**F**, **K**–**L**). *P* values that were less than 0.2 were indicated. *n* = 3 in WT group, *n* = 10 in Ctrl_AD and Inulin_AD groups, represented by a dot in the graphs. Data were presented in mean ± SD. **M** Western blot and quantification of IBA1 level in the forebrain region of WT, Ctrl_AD, and Inulin_AD mice. Unpaired *t*-test was used to determine statistical significance between Ctrl_AD and Inulin_AD groups. *n* = 3 in WT group, *n* = 5 in Ctrl_AD and Inulin_AD groups, represented by a dot in the graphs. Data were presented in mean ± SD. Supporting data values for **D**–**F**, **J**–**K**, **L**–**M** were included in Additional file 6: Supporting data values. Original images of **A**–**C**, **G**–**I**, **M** were included in Additional file 7: original images

**Fig. 10:**
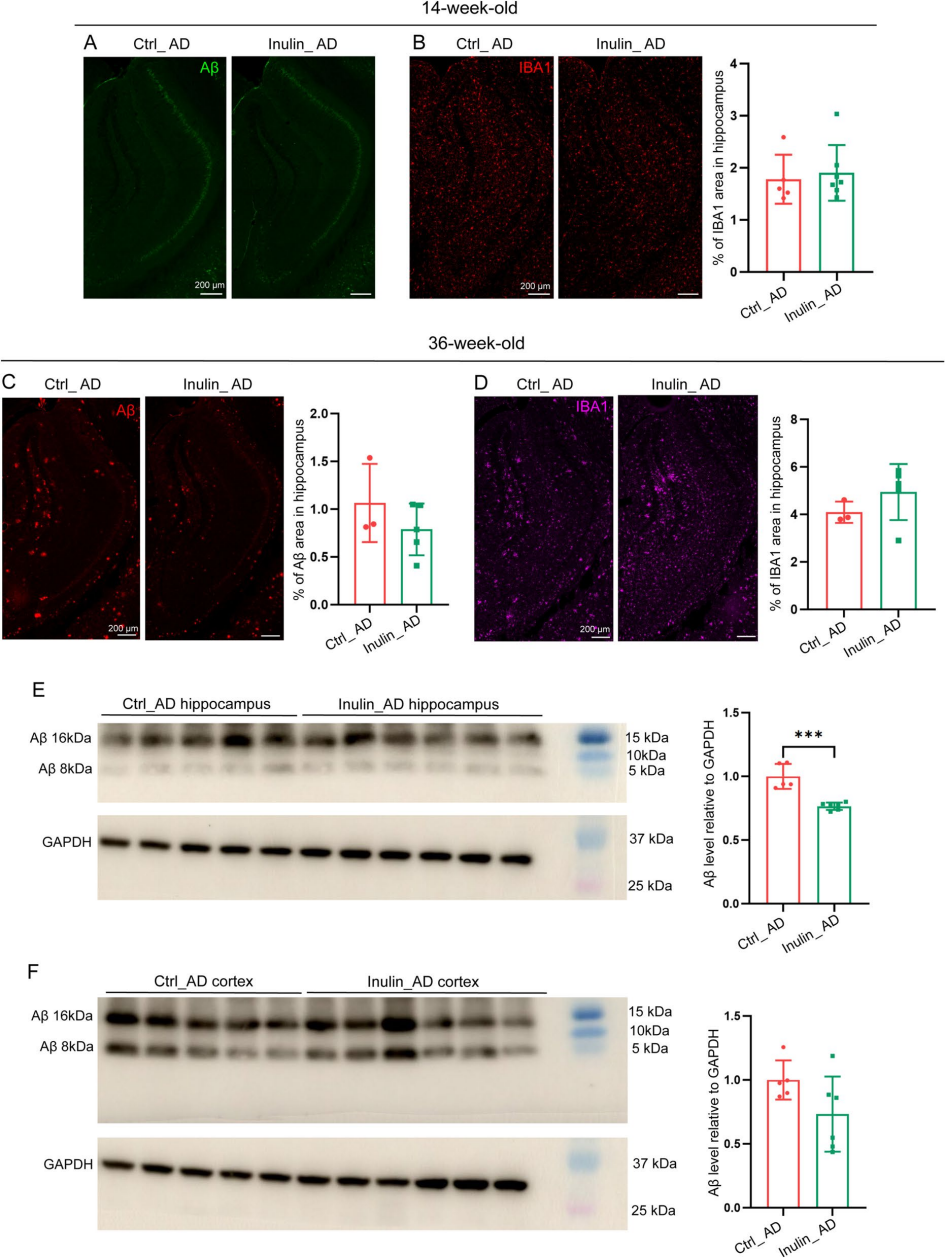
Prolonged dietary inulin supplementation reduced Aβ plaque burden in hippocampus. **A** Illustration of Aβ plaque deposition in hippocampus of Ctrl_AD and Inulin_AD animals at 14-week-old. Scale bar, 200 µm. Illustration and quantification of Aβ plaque deposition in 14- (**B**) and 36-week-old hippocampus (**C**), and microgliosis in 36-week-old hippocampus (**D**). 6E10 and IBA1 staining was performed on the same section in **C**–**D**. Unpaired *t*-test was used to determine statistical significance. *, *P* < 0.05. *n* = 3 in Ctrl_AD group, *n* = 5 in Inulin_AD group, represented by a dot in the graphs. Data were presented in mean ± SD. Scale bar, 200 µm. Western blots and quantification of Aβ level in hippocampus (**E**) and cortex (**F**) of Ctrl_AD and Inulin_AD mice. Intensity of Aβ 16 and 8 kDa bands was added for quantification. Unpaired *t*-test was used to determine statistical significance. ***, *P* < 0.001. *n* = 5 in Ctrl_AD group, *n* = 6 in Inulin_AD group, represented by a dot in the graphs. Data were presented in mean ± SD. Supporting data values for **B**–**F** were included in Additional file 6: Supporting data values. Original images of **A**–**F** were included in Additional file 7: original images

Using GFAP as a marker for reactive astrocytes to assess astrogliosis, we observed a significant reduction of astrogliosis in caudal part of the ventral cortex of Inulin_AD animals that mainly covers the entorhinal area, postpiriform transition area, and endopiriform nucleus (Fig. 8H–J), but not any other regions (F ig. 8A–I, K–L) or the forebrain homogenate (Fig. 8M). Using IBA1 as a pan-microglial marker, a non-significant decreasing trend of IBA1 area was observed in all assessed brain regions of Inulin_AD animals (Fig. 9A–L). In line with such trend, the overall IBA1 protein level in the forebrain homogenate was significantly reduced in the Inulin_AD animals (Fig. 9M). Given such reducing trend in Aβ plaque burden and microgliosis, we further examined these parameters in 14- and 36-week-old Ctrl_AD and Inulin_AD animals. With a very low level of Aβ plaque burden in both groups at the 14-week time point, no difference was observed for microgliosis (Fig. 10A–B). At the 36-week time point, Aβ plaque burden showed significant reduction in the hippocampus homogenate of Inulin_AD animals (Fig. 10E), although only a trend was observed on the tissue sections (Fig. 10C). No difference was observed for microgliosis (Fig. 10D). Cortex homogenate of Inulin_AD animals also showed a non-significant decreasing trend of Aβ plaque burden (Fig. 10F).

## Discussion

Emerging evidence has suggested that AD onset and progression are associated with dietary patterns [23]. While high caloric Western diet is a risk factor for AD development, plant-based diet correlates with mild and moderate AD [24] and reduction in AD biomarkers, including β-amyloid plaques and tau tangles [25]. In the present study, we divided the brain of 5xFAD mice into different regions and conducted snRNA-seq analysis to explore the impact of dietary inulin supplementation on cellular composition and gene transcription at the whole genome level. In the forebrain region, inulin induced an overall decrease in microglia proportion, which is consistent with a tendency to reduced IBA1 staining in many regions of the cortex and hippocampus. This phenomenon might be related to the reported anti-inflammatory effects of inulin in AD mouse models [18, 26]. In the interbrain region that includes amygdala, the proportion of the *Prkcd*^+^ CeA neurons was found to be elevated upon inulin supplementation. These GABAergic neurons have been reported to suppress food intake [27] and reduce conditioned fear [28], which may account for the tendency to reduced body weight in animals fed with inulin-rich diet, as well as the enrichment in the subpopulation of metabolism-related astrocytes in the same region. In cerebellum, a granule cell subpopulation that expressed high levels of *Rasgrf1*, a gene that has been shown to be specifically expressed in the anterior lobe of cerebellum [29], had a tendency to convert into a *Rasgrf1*^−^ subpopulation upon inulin supplementation, suggesting an inulin-induced regional rearrangement of the granule cells.

Earlier bulk RNA sequencing of ALDH1L1-positive astrocytes from adult mouse brain demonstrated their functional heterogeneity among cortex, hippocampus, caudate-putamen, nucleus accumbens, thalamus, and hypothalamus [30]. A region-specific aging response of astrocytes has also been shown in visual cortex, motor cortex, cerebellum, and hypothalamus of 4-month-old adult and 2-year-old aged mice [31]. Recently, a spatial atlas of all cell types in the entire mouse brain revealed astrocyte subclasses that are specific to the olfactory, telencephalon, and cerebellum [32]. Our results are in line with these previous studies, demonstrating the presence of region-specific astrocyte subtypes. Such phenomenon may be explained by the regional specification for astrocyte-to-neuron reprogramming as previously described [33]. In contrast, microglia subtypes were more evenly distributed among the forebrain, interbrain, and brainstem regions, which agrees with a previous deep scRNA-seq and region-specific bulk RNA-seq analysis showing that the *Tmem119*^+^ homeostatic microglia in the adult mouse brain shows limited transcriptomic heterogeneity across different brain regions [34]. However, other studies have reported the existence of microglia diversity among cerebellum, cerebral cortex, hippocampus, and striatum [35], as well as region-specific phenotypes of microglia within the basal ganglia [36]. These studies were all conducted in WT mice of different ages, therefore representing more of the microglia at the resting state. In AD mice, a large proportion of the microglia population becomes activated, which might account for low heterogeneity that we observed in these mice.

*Akkermansia muciniphila* is the only species of the Verrucomicrobiota phylum in human intestine that solely relies on mucus protein as its energy source [37]. *Akkermansia* abundance has been shown to be significantly decreased in 8-month-old Aβ precursor protein (APP)/presenilin 1 (PS1) mice [38]. In line with this, daily feeding of APP/PS1 mice with *Akkermansia muciniphila* for 6 months led to decreased Aβ plaque burden in cerebral cortex and improved performance in the Y-maze test [39]. Similarly, oral treatment with *Akkermansia muciniphila* in AD-like rat models reduced Aβ deposition in cortex and alleviated cognitive impairment in the Morris water maze test [40]. On the other hand, several studies analyzing the gut microbiota of AD patients revealed increased *Akkermansia muciniphila* abundance compared to healthy people [41–43], indicating a negative role of this bacteria species on AD progression. Dietary supplementation of different types of fiber has differential effects on *Akkermansia muciniphila* abundance in the gut [44]. Six weeks of daily inulin treatment in C57BL/6 J mice can significantly elevate the percentage of *Akkermansia muciniphila* in fecal pellets [45]. In the present study, we did not observe any changes in *Akkermansia muciniphila* abundance when comparing WT animals fed with control or inulin-rich diet, or WT and AD animals that were both fed with control diet. In fact, the level of *Akkermansia muciniphila* was barely detected in these three groups. When AD animals were fed with inulin-rich diet, the abundance of *Akkermansia muciniphila* erupted to occupy more than 10% of the total gut microbiota. These results suggest that *Akkermansia muciniphila* growth requires the presence of both AD pathology and inulin enrichment. Our findings agree with a previous prospective clinical study that a modified Mediterranean-ketogenic diet could increase gut *Akkermansia* abundance in patients with mild cognitive impairment [46]. AD pathology [47] and inulin enrichment [48] have been shown to stimulate mucin synthesis and promote mucin secretion in the ileum, respectively, which might explain the mechanism underlying the synergized effect of the two factors on *Akkermansia muciniphila* abundance.

APP/PS1 transgenic mice fed with diet supplemented with *A. tequilana*-derived fructans between 4- and 6-month-old of age showed reduced number of Aβ plaques and GFAP^+^ astrocytes in hippocampus, as well as improved short-term working memory, cognition, and spatial memory [49]. However, we only observed marginal effects of inulin supplementation on astrogliosis and microgliosis, and no changes on Aβ plaque burden. Such discrepancy may be explained by the different AD mouse models and experimental setups that were used. The 5xFAD mouse model used in the present study carries a human APP transgene harboring three FAD mutations and a human PS1 transgene harboring two FAD mutations, both of which are driven by the neural-specific *Thy1* promoter element [20], while the APP/PS1 transgenic mice carries a chimeric mouse/human APP transgene harboring two Swedish mutations that are different from those carried by 5xFAD mice and a human PS1 transgene harboring the FAD associated exon-9-deleted variant, both of which are driven by the mouse prion protein promoter element [50]. Notably, neuropathology and cognitive impairment are both more severe and show an earlier onset in 5xFAD mice compared to APP/PS1 mice. Moreover, a previous study has shown that the two AD mouse models behave differently in memory, affective behavior, and neuropathology [51]. In line with our findings, a recent study assessed the impact of dietary supplementation with high acetate and butyrate on neuropathology of the 5xFAD mice and found no difference from the control diet group in terms of Aβ plaque size and area, as well as level of microglia activation [52].

Over the past decade, the role of brain-gut interactions mediated by gut microbiota-derived metabolites has gained increasing attention in the context of AD development and progression. Gut microbiome of patients with dementia due to AD has been shown to have decreased microbial diversity and greatly altered composition compared to control age- and sex-matched individuals [53]. Therapeutic strategies for alleviation of AD pathology by restoring a balanced gut microbial community in AD patients have been widely explored, including fecal microbiota transplantation, introduction of beneficial bacteria, and antibiotics treatment [54]. In addition, growing evidence has indicated a correlation between plant-based food adherence and protection against cognitive decline, although a cause-and-effect relation is yet to be established [55]. Here, we show that dietary inulin supplementation in 5xFAD mice has limited effects on Aβ plaque burden, astrogliosis, microgliosis, and spatial memory. These findings indicate that the addition of a single type of dietary fiber to the daily diet is insufficient to significantly impact AD pathology. Instead, a more diversified approach to fiber supplementation may be necessary to achieve meaningful therapeutic benefits. This highlights the complexity of gut-brain interactions in AD and underscores the need for further research into multifaceted dietary interventions.

To include all cell types in the present study, snRNA-seq method was used to accommodate the size of the neuronal cells. However, such exclusive collection of nuclear transcripts prevents assessment of the transcripts that have already been transported into the cytoplasm. A previous study reported that snRNA-seq has low sensitivity to detect microglial activation genes in human brains [56]. It is therefore possible that certain specific characteristics of the microglia population may have been missed in our study due to this limitation. Cholesterol metabolism has been implicated in AD pathogenesis since the initial description of the disease by Alois Alzheimer. In our snRNA-seq data, cholesterol biosynthesis was the top downregulated pathway in the neurons and astrocytes of Inulin_AD animals. In addition, the protein level of HMGCR was significantly reduced in the forebrain homogenate, suggesting a negative correlation between inulin intake and cholesterol biosynthesis. Future studies should focus on elucidating the underlying mechanisms by which inulin modulates cholesterol metabolism and its implications for AD pathology. Here, we applied inulin-rich diet right after sexual maturation, a time point that Aβ plaques remain undetectable. Investigating the cellular effects of diet switch at early, middle, or late stages of AD progression will provide more comprehensive indications for future dietary guidance for AD patients. Moreover, we did not find marker genes that were specifically expressed in astrocyte subpopulations that demonstrated proportional alterations upon inulin supplementation. Such technical difficulties prevent us from isolating specific astrocyte subpopulations for further analysis. Furthermore, feeding 14-week-old 5xFAD mice with *Akkermansia muciniphila* for 4 weeks only altered the level of microgliosis, but not the Aβ plaque burden or level of astrogliosis. Feeding the bacteria during later stages of AD progression will provide further insights on the causal relationship between *Akkermansia muciniphila* abundance and AD pathology.

Nevertheless, our comprehensive single-cell transcriptomic atlas of the entire AD mouse brain in response to high fiber intake by sequencing three independent biological replicates per diet group is the first of its kind and represents a unique resource. In the majority of transcriptomic studies at single cell level, cells are pooled from different animals for analysis and therefore individual variance is often missed, preventing robust statistical assessment of differences between treatment groups. Indeed, we did observe variations in the proportion of major cell types and cell subtypes among the three replicates, which highlights the importance in considering using biological replicates for future high throughput investigations.

## Conclusions

By generating a comprehensive atlas on the high-fiber-induced transcriptomic changes in individual cells of the entire mouse brain, our results pave the way for future studies aiming to link fiber-induced intestinal microbiome changes with cellular changes in the brain of AD, as well as other neurodegeneration diseases.

## Methods

### Animals

Mouse experiments were carried out in accordance with the protocol approved by the Institutional Animal Care and Use Committee (IACUC) of Chinese Institute for Brain Research (CIBR-IACUC-035) and Peking University (Psych-XieM-2). Mice were housed under a regular 12-h day/night cycle with ad libitum access to food and water. 5xFAD mice were purchased from the Jackson Laboratory (JAX: 034840). All mice used in the present study were male. AIN93G, AIN93M, and AIN93M supplemented with 7.5% long-chain inulin (Ararat, CNE60307) diets were purchased from Jiangsu Xietong Pharmaceutical Bio-engineering Co., Ltd. Inulin was sourced from Chicory with a 98% purity.

### 16S rRNA microbiome sequencing

Fecal pellets were collected at the end of the feeding study when mice were 29–30-week-old and stored at − 80 °C until DNA extraction. DNA was extracted with Magnetic Soil and Stool DNA Kit (TianGen, Cat #DP712), followed by running on a 2% agarose gel for band detection at around 310 bp. The bacterial hypervariable V4 region of 16S rRNA was amplified using primers 515 F (GTGCCAGCMGCCGCGGTAA) and 806R (GGACTACHVGGGTWTCTAAT) with barcode. The polymerase chain reaction (PCR) was conducted with the Phusion® High-Fidelity PCR Master Mix (New England Biolabs). Purified PCR products plus indexes were used to build the sequencing libraries using the NEB Next® Ultra™ II FS DNA PCR- free Library Prep Kit (New England Biolabs, Cat #E7430L). Libraries were validated with Qubit and real-time qPCR, before sequencing with the NovaSeq 6000 system (Illumina). High-quality paired-end reads were assigned to samples based on their unique barcode and truncated, then merged using FLASH (Fast Length Adjustment of Short reads, v1.2.11) [57]. Data filtering on raw tags were performed using the fastp (version 0.23.1) [58]. Chimera sequences were detected by comparing the tags with the Silva database (16S/18S) and were subsequently removed using vsearch (version 2.16.0) [59]. Denoise and species annotation were performed with QIIME2 (version QIIME2-202,202). Absolute abundance of amplicon sequence variants was normalized to the sequence number of the sample with the least reads and was subsequently applied for alpha and beta diversity analysis. Cluster analysis was performed by principal component analysis, which was applied to reduce the dimension of the original variables using the ade4 and ggplot2 package in R (version 4.0.3).

*Akkermansia muciniphila* feeding.

Male 5xFAD mice were divided into two groups: the Akk_AD group that received oral gavages of 1 ml bacterial suspension containing 2 × 10^9^ CFU of *Akkermansia muciniphila* (Bio-Sci Bio, China) and the Vehicle_AD group that received only the medium. The experiment commenced at 14 weeks of age, with treatments administered twice weekly for 4 weeks. Mice were euthanized and tissues harvested at 18 weeks of age. The bacterial solution was freshly prepared on a weekly basis to ensure viability.

### Tissue dissection and preparation for snRNA-seq

Mice were sacrificed by cervical dislocation sterilized with 75% ethanol. The entire brain was placed in cold sterile PBS solution right after dissection. One hemisphere of the brain was further dissected into forebrain, interbrain, brainstem, and cerebellum as shown in Fig. S1 A. Interbrain was separated from the brainstem along the boundary of thalamus and midbrain. Samples of different brain regions were snap frozen in liquid nitrogen right after separation and stored at − 80 °C until further processing.

Frozen brain tissues were lysed in Hibernate A®/B27®/GlutaMAX™ (HEB) medium (Gibco) plus lysis buffer containing 10 mM Tris–HCL pH 7.4, 10 mM NaCl, 3 mM MaCl_2_, and 0.1% NP-40 for 15 min on ice, before filtering with a 30 µm MACS® SmartStrainer (Miltenyi Biotec). Nuclei suspension was collected after serial centrifuges at 500 rcf at 4 °C and washes with Nuclei Wash and Resuspension Buffer containing 1% BSA and 0.2 U/µl RNase inhibitor. Myelin was removed by incubating the nuclei suspensions with Myelin Removal Beads II and LS column (Miltenyi Biotec). Density gradient centrifugation with sucrose cushion buffer was performed to concentrate the isolated nuclei. Once the nuclei concentration reached 1000 nuclei/µl, the samples were immediately proceeded for library preparation.

### snRNA-seq library preparation

Nuclei suspensions were loaded onto Chromium microfluidic chips with 3′ v3 chemistry and barcoded with a 10 × Chromium Controller. RNA from the barcoded cells was subsequently reverse-transcribed to construct the sequencing libraries with the Chromium Single Cell 3′ v3 reagent kit (10 × Genomics) according to manufacturer’s instruction. Sequencing was performed by the NovoSeq X plus system (Illumina) with 150-bp paired-end reads.

### snRNA-seq data analysis

Demultiplexed fastq files were aligned to mouse reference genome mm10 using CellRanger (version 7.1.0) with introns included. Background removal was performed with CellBender (version 0.2.2). Sequencing data were analyzed using Seurat package (version 4.3.0.1) [60] in R (version 4.2.2). Nuclei that qualified the following criteria were selected for further analysis: 500 ~ 6000 identified genes, 1000 ~ 50,000 unique molecular identifiers, and less than 0.2% mitochondrial genes. In order to eliminate the effect of mitochondrial genes on subsequent re-clustering and differential expression gene analysis, nuclei with mitochondrial genes > 0.2% were removed. Doublets were removed using the *DoubletFinder* function (version 2.0.3) [61] for each sample. Top 8% of the nuclei that had the highest doublet score were excluded from further analysis.

Each sample was log-normalized with a scale factor equals to 10,000. The top 2000 variable features were identified using the *FindVariableFeatures* function. Data integration was performed using the *SelectIntegrationFeatures*, *FindIntegrationAnchors*, and *IntegrateData* functions. Then, the integrated matrices were scaled with PCA and the top 15 principal components were kept to project into a two-dimensional space using Uniform Manifold Approximation and Projection (UMAP). K-nearest neighbor algorithm was performed and Louvain algorithm was applied for cell clustering. Clustering resolution of 0.1 was used for all analysis. Cell type annotation was performed according to previously identified marker genes [29, 62–64] and genes listed in http://bio-bigdata.hrbmu.edu.cn/CellMarker/index.html. Clusters expressed more than one type of marker genes were annotated as unknown. Re-clustering of each cell type was performed with the *subset* function.

Marker genes of each cluster were identified by the *FindAllMarkers* function with the following parameters: at least 25% of the cells expressed the genes and a log2 fold change (log2 FC) greater than 0.25. The *EnrichGO* and *compareCluster* functions of the clusterProfiler (version 4.6.2) were used for GO biological process pathway analysis using the species-specific annotation database (org.Mm.eg.db). An adjusted *P* value was obtained using the Benjamin and Hochberg FDR method. Expression of genes in upregulated GO pathway had log2 FC > 0.3 and p.adj < 0.05. Expression of genes in downregulated GO pathway had log2 FC <  − 0.3 and p.adj < 0.05. Wilcoxon-based statistic was used for the *FindAllMarkers* function. For other multiple testing corrections for snRNA-seq analysis, default parameters in the function were used. For cell proportional analysis, two-way ANOVA with Sidak’s multiple comparisons test was used.

### RNA velocity analysis

The spliced and un-spliced counts were calculated using velocyto (version 0.17.17) [22] and saved in the loom format. Subsequently, the count matrix and metadata were extracted from the Seurat object in R and imported into the python environment. RNA velocities were estimated using scVelo (version 0.3.2) [65]. Initially, moments of the gene expression distribution were computed by the *moments* function, then the dynamic components of gene expression were captured using the *recover_dynamics* function. Velocity vectors were inferred using a gene expression-based method through the velocity function. The velocity graph was constructed to visualize transitions between cell clusters using the *velocity_embedding_stream* function.

### Immunostaining

Mice were anesthetized with isoflurane and perfused with 1xPBS through heart until the liver and kidneys turned completely white. Then, the brain was dissected out and separated into two hemispheres along the medial sagittal plane. Each hemisphere was fixed in freshly prepared and pre-cooled 4% paraformaldehyde (PFA) with rotation at 4 °C for overnight. Samples were sequentially dehydrated through 20% and 30% sucrose solutions, before embedding in OCT solution (Tissue-Tek) and storing at − 80 °C. Samples were sectioned into 14-µm-thick sections using a CryoStar NX70 HOMP cryostat (Thermo scientific). Tissue sections were blocked in blocking buffer containing 5% donkey serum and 0.2% Triton X-100 for 15 min at room temperature before incubating with the primary antibody overnight at 4 °C. After washing with PBS, secondary antibody was applied for 1 h at room temperature. Sections were imaged with a Virtual Slide Microscope (Olympus, VS120-S6-W). Following antibodies were used: Aβ6E10, 1:1000 (Biolegend, Cat #803,002); GFAP, 1:500 (Abcam, Cat #ab7260); IBA1, 1:500 (Wako, Cat #019–19741); AQP4, 1:100 (Santa Cruz, Cat#16,473–1-AP); CLU, 1:20 (Thermo, Cat#PA5-46,931); donkey anti-mouse secondary antibody 1:1000 (Invitrogen, Cat #A21202); donkey anti-rabbit secondary antibody 1:1000 (Invitrogen, Cat #A31572); and donkey anti-goat secondary antibody 1:1000 (Invitrogen, Cat #A11055). Immunostaining measurements were performed with ImageJ (version 1.54).

### Western blot

Brain tissues were sonicated and lysed using RIPA buffer supplemented with protease and phosphatase inhibitors. The homogenates were incubated on ice for 30 min and subsequently centrifuged at 12,000 rpm for 10 min at 4 °C. The extracted proteins were mixed with 4 × loading buffer (Laemmli buffer/β-mercaptoethanol = 9/1) and boiled at 98 °C for 5 min prior to electrophoresis on polyacrylamide gels. Proteins were separated using 10% or 12% polyacrylamide gels, with 12% gels reserved for oligomer analysis. Following electrophoresis, proteins were transferred onto polyvinylidene fluoride membranes. The membranes were blocked with 3% non-fat milk in TBST (0.1% Tween-20) and then incubated overnight at 4 °C in 1% non-fat milk. Immunoreactivity was detected using horseradish peroxidase (HRP)-conjugated secondary antibodies. To re-probe the membranes, bound primary and secondary antibodies were removed using a stripping buffer (Thermo; #46,430), followed by incubation with a GAPDH antibody. Immunoblots were developed using the Immobilon Western Chemiluminescent HRP Substrate (Millipore) and visualized using an Amersham ImageQuant 800 system. Quantification was performed using ImageJ software.

### Barnes maze test

The mice’s circadian rhythms were synchronized 1 week prior to the Barnes maze test. The Barnes maze consists of a white circular platform with a diameter of 90 cm, containing 20 evenly spaced holes along its perimeter. A bright white light source was positioned directly above the platform during the experiment. Each mouse was initially placed in the center of the maze within a black chamber, which was removed after 5 s. During the training sessions, the mouse was allowed to freely explore the maze until it entered the escape tunnel or for a maximum of 3 min. The escape tunnel was consistently placed under the same hole, and the spatial cues remained unchanged throughout the experiment. Mice underwent one training session per day for 5 consecutive days. For the test sessions, the escape tunnel was removed, and the mouse was allowed to explore the maze freely for 3 min to evaluate spatial memory. The short-term memory test was conducted 3 h after the final training session, with the time spent in the target quadrant recorded as the primary measure. The entire process was captured on video and analyzed using EthoVision XT software (version 16) from Noldus.

### Statistical analysis

Two-way ANOVA or unpaired *t*-tests were used to assess statistical significance by GraphPad Prism 8.0.2 as specified in the corresponding figure legends. Results were presented as mean ± standard deviation (SD) in all analysis. A *P* value less than 0.05 was considered as statistically significant. *P* values that were less than 0.2 were indicated in all graphs.

## Supplementary Information

Additional file 1: Table S1 Compositions of the Ctrl and inulin diet. To maintain the same calorie between the two diets, composition of starch corn was slightly reduced to accommodate the calorie of added long-chain inulin in the inulin diet

Additional file 2: Fig. S1 Brain region definition and quality control of snRNA-seq analysis.Photos of the medial sagittal plane of the mouse brain showing the definition of different brain regions used for snRNA-seq. FB, forebrain; IN, interbrain; BS, brainstem; CB, cerebellum.Violin plots showing UMI count, number of detected genes, and percentage of sequencing reads from mitochondria of snRNA-seq data from the forebrain region, interbrain region, brainstem, and cerebellum

Additional file 3: Fig. S2 Analysis of the astrocyte, oligodendrocyte, and OPC populations in the forebrain region.UMAP of neuron, microglia, astrocyte, oligodendrocyte, and OPCin the forebrain region of Ctrl_AD and Inulin_AD mice.Violin plot of marker gene expression level of neuron, microglia, astrocyte, oligodendrocytes, and OPCs.Cell proportion comparison of neuron, microglia, astrocyte, oligodendrocytes, and OPCsbetween the Ctrl_AD and Inulin_AD groups. Each dot represents one independently sequenced mouse. Two-way ANOVA with Sidak’s multiple comparisons test was used to determine statistical significance. Data were presented as mean ± SD

Additional file 4: Fig. S3 Analysis of the microglia, oligodendrocyte, and OPC populations in the interbrain region.UMAP of microglia, oligodendrocytes, and OPCsin the forebrain region of Ctrl_AD and Inulin_AD mice.Violin plot of marker gene expression level of microglia, oligodendrocytes, and OPCs.Cell proportion comparison of microglia, oligodendrocytes, and OPCsbetween the Ctrl_AD and Inulin_AD groups. Each dot represented one independently sequenced mouse. Two-way ANOVA with Sidak’s multiple comparisons test was used to determine statistical significance. Data were presented in mean ± SD

Additional file 5: Fig. S4 Analysis of the microglia, oligodendrocyte, and OPC populations in brainstem.Illustrating images of AQP4 and CLU immunostaining in Ctrl_AD and Inulin_AD animals. Scale bar, 50 µm. Original images were included in Additional file 7: original images.UMAP of neuron, microglia, oligodendrocytes, and OPCsin brainstem of Ctrl_AD and Inulin_AD mice.Violin plot of marker gene expression level of neuron, microglia, oligodendrocytes, and OPCs.Cell proportion comparison of neuro, microglia, oligodendrocytes, and OPCsbetween the Ctrl_AD and Inulin_AD groups. Each dot represented one independently sequenced mouse. Two-way ANOVA with Sidak’s multiple comparisons test was used to determine statistical significance. Data were presented in mean ± SD

Additional file 6: Supporting data values. File contains raw data for quantifications in Fig. 1B–J, L–O; Fig. 2D–G, I, K; Fig. 3E–H; Fig. 4D–E; Fig. 7D–F, J–K, L–O; Fig. 8D–F, J–K, L–M; Fig. 9D–F, J–K, L–M; Fig. 10B–F

Additional file 7: Original images. File contains original images for Fig. 1 K; Fig. 2D–G, I, K; Fig. 3D–H; Fig. 4D–E; Fig. 7 A–C, G–I, M; Fig. 8 A–C, G–I, M; Fig. 9 A–C, G–I, M; Fig. 10 A–F

## Data Availability

All data generated or analysed during this study are included in this published article, its supplementary information files and publicly available repositories. All raw sequencing data reported in the present study were deposited in the Gene Expression Omnibus (GEO) database under accession number GSE262881. All codes for snRNA-seq data analysis were in the GitHub repository: https://github.com/XiaoyanWang11/snRNA-seq-of-inulin_-Diet_FAD, and Zenodo: 10.5281/zenodo.15289277.

## Abbreviations

AD: Alzheimer’s disease
FAD: 5X Familial AD
Aβ: Amyloid-β
CNS: Central nervous system
IBA1: Calcium-binding adaptor molecule 1
GFAP: Glial fibrillar acidic protein
PCA: Principal component analysis
OPCs: Oligodendrocyte progenitor cells
sc- and sn-RNAseq: Single-cell and single-nucleus RNA sequencing
WT: Wildtype
GSEA: Gene Set Enrichment Analysis
DEGs: Differentially expressed genes
CeA: Central nucleus of the amygdala
UMAP: Uniform Manifold Approximation and Projection
log2 FC: log2 fold change
PFA: Paraformaldehyde
HRP: Horseradish peroxidase
SD: Standard deviation
