# Supplementary figures and images for: A single-cell transcriptomic atlas of all cell types in the brain of 5xFAD Alzheimer mice in response to dietary inulin supplementation
Source: BMC Biol. 2025 May 9;23:124. doi: 10.1186/s12915-025-02230-x (PMC12065180; doi:10.1186/s12915-025-02230-x)

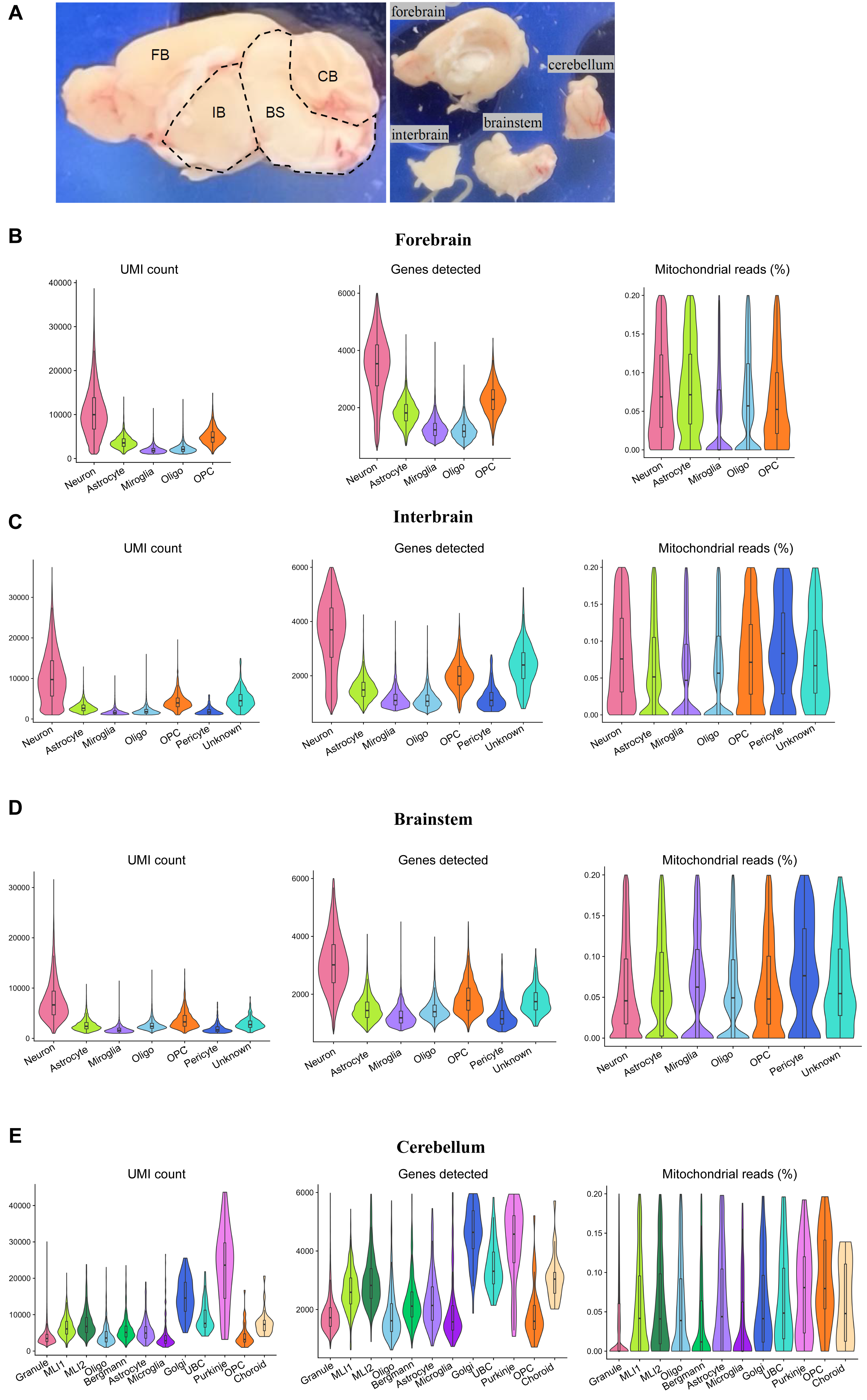

Supplement: Supplementary file 2 — Additional file 2: Fig. S1 Brain region definition and quality control of snRNA-seq analysis.Photos of the medial sagittal plane of the mouse brain showing the definition of different brain regions used for snRNA-seq. FB, forebrain; IN, interbrain; BS, brainstem; CB, cerebellum.Violin plots showing UMI count, number of detected genes, and percentage of sequencing reads from mitochondria of snRNA-seq data from the forebrain region, interbrain region, brainstem, and cerebellum [file 12915_2025_2230_MOESM2_ESM.tif]

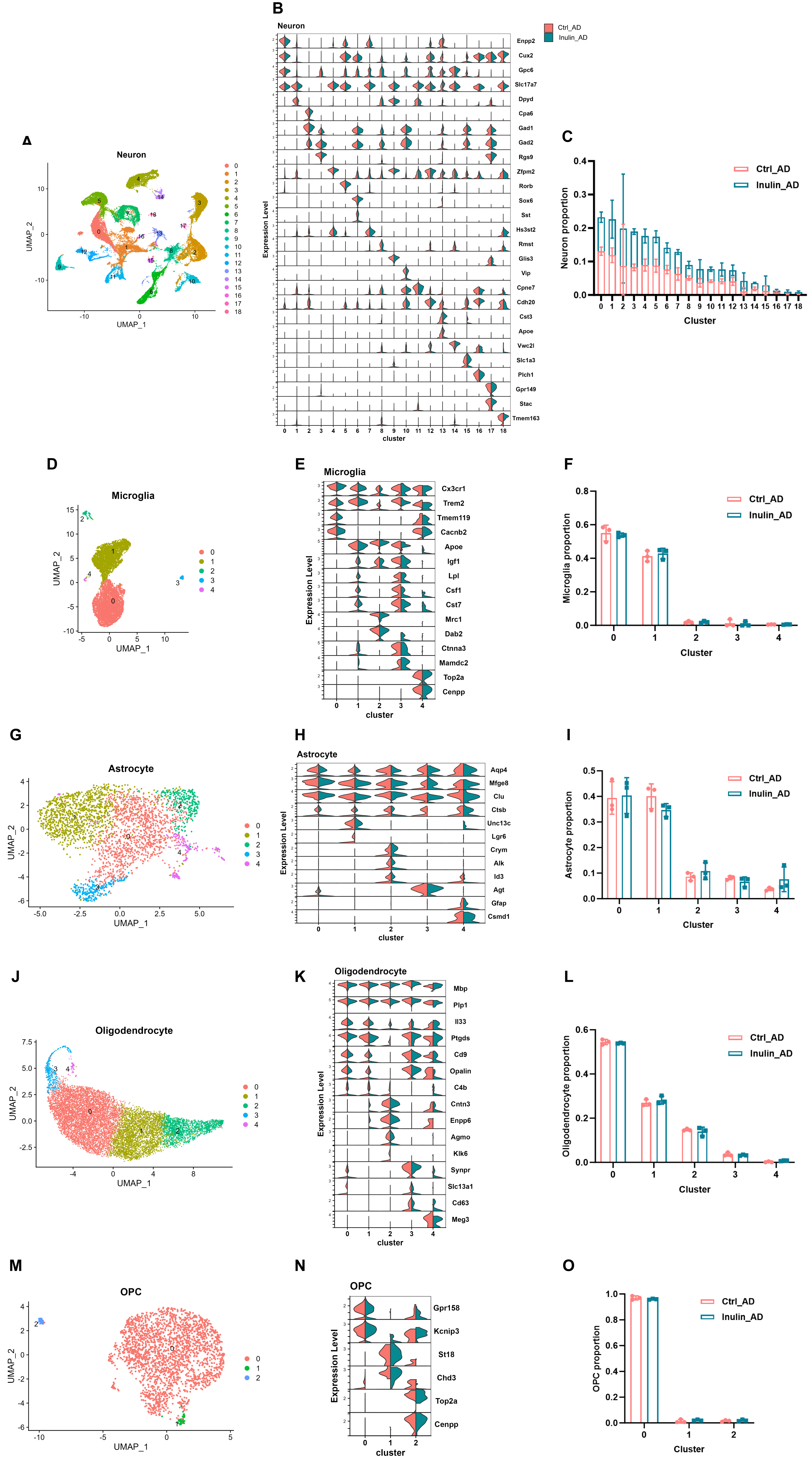

Supplement: Supplementary file 3 — Additional file 3: Fig. S2 Analysis of the astrocyte, oligodendrocyte, and OPC populations in the forebrain region.UMAP of neuron, microglia, astrocyte, oligodendrocyte, and OPCin the forebrain region of Ctrl_AD and Inulin_AD mice.Violin plot of marker gene expression level of neuron, microglia, astrocyte, oligodendrocytes, and OPCs.Cell proportion comparison of neuron, microglia, astrocyte, oligodendrocytes, and OPCsbetween the Ctrl_AD and Inulin_AD groups. Each dot represents one independently sequenced mouse. Two-way ANOVA with Sidak’s multiple comparisons test was used to determine statistical significance. Data were presented as mean ± SD [file 12915_2025_2230_MOESM3_ESM.tif]

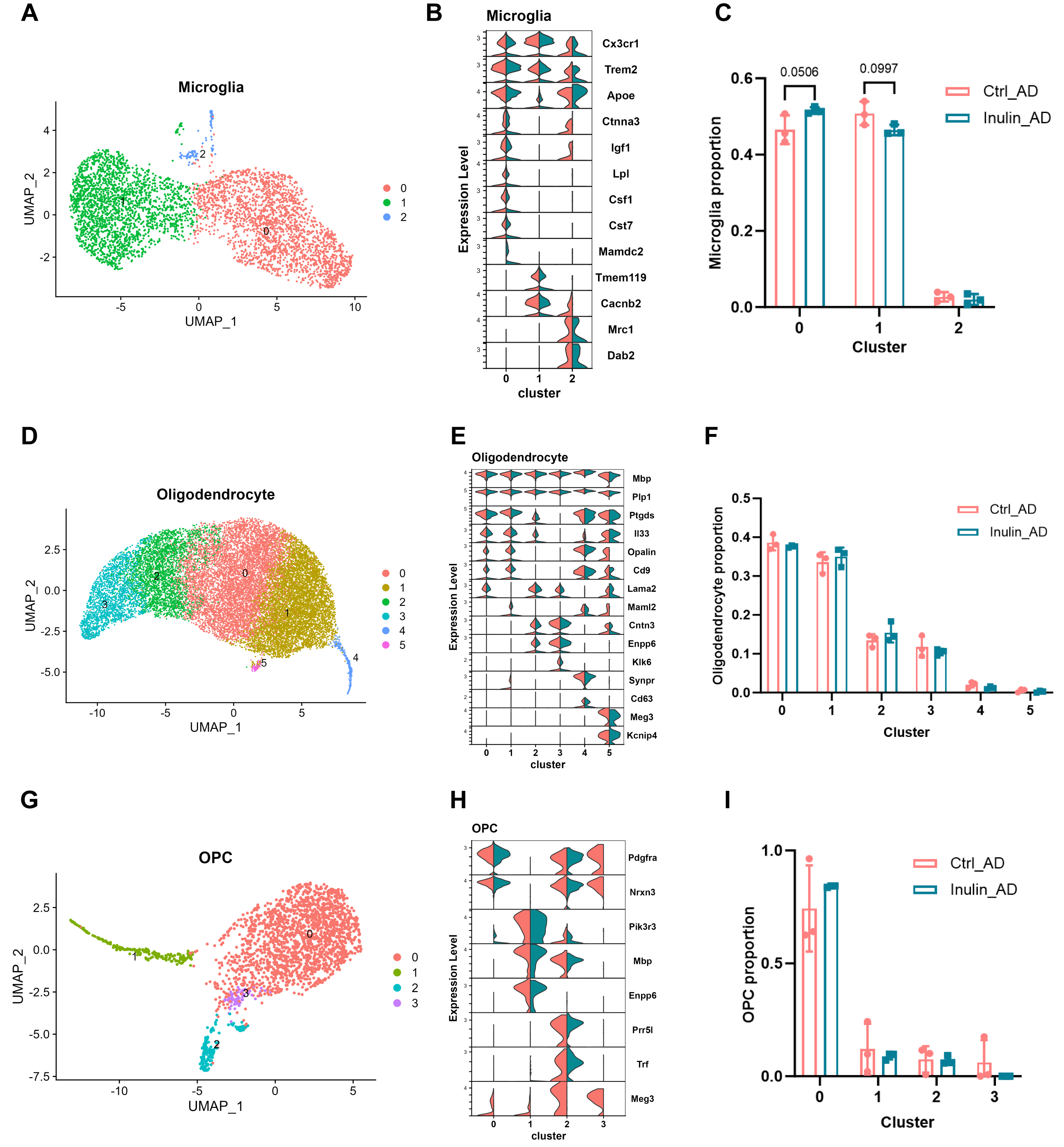

Supplement: Supplementary file 4 — Additional file 4: Fig. S3 Analysis of the microglia, oligodendrocyte, and OPC populations in the interbrain region.UMAP of microglia, oligodendrocytes, and OPCsin the forebrain region of Ctrl_AD and Inulin_AD mice.Violin plot of marker gene expression level of microglia, oligodendrocytes, and OPCs.Cell proportion comparison of microglia, oligodendrocytes, and OPCsbetween the Ctrl_AD and Inulin_AD groups. Each dot represented one independently sequenced mouse. Two-way ANOVA with Sidak’s multiple comparisons test was used to determine statistical significance. Data were presented in mean ± SD [file 12915_2025_2230_MOESM4_ESM.tif]

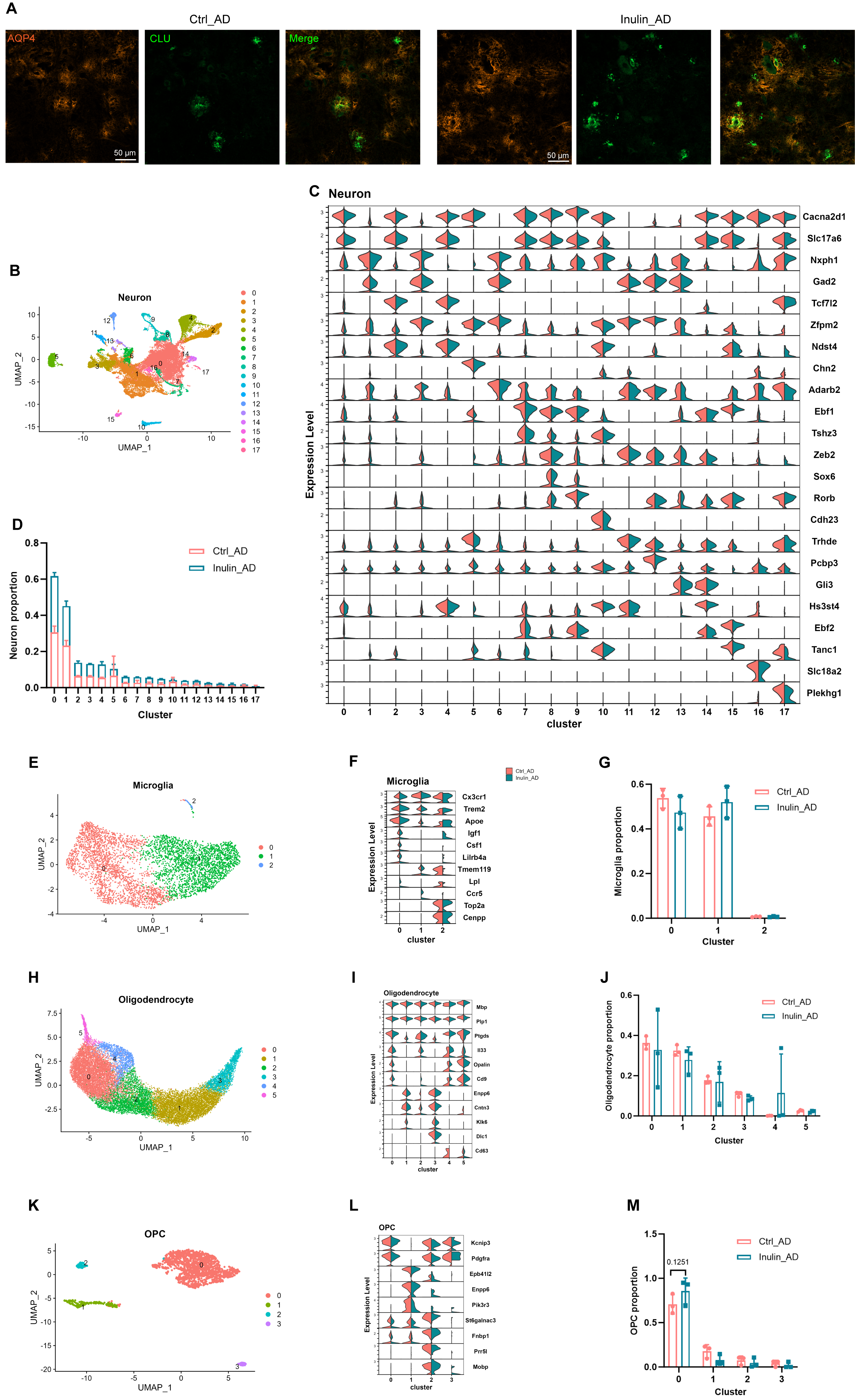

Supplement: Supplementary file 5 — Additional file 5: Fig. S4 Analysis of the microglia, oligodendrocyte, and OPC populations in brainstem.Illustrating images of AQP4 and CLU immunostaining in Ctrl_AD and Inulin_AD animals. Scale bar, 50 µm. Original images were included in Additional file 7: original images.UMAP of neuron, microglia, oligodendrocytes, and OPCsin brainstem of Ctrl_AD and Inulin_AD mice.Violin plot of marker gene expression level of neuron, microglia, oligodendrocytes, and OPCs.Cell proportion comparison of neuro, microglia, oligodendrocytes, and OPCsbetween the Ctrl_AD and Inulin_AD groups. Each dot represented one independently sequenced mouse. Two-way ANOVA with Sidak’s multiple comparisons test was used to determine statistical significance. Data were presented in mean ± SD [file 12915_2025_2230_MOESM5_ESM.tif]
